# Novel Benzo[*a*]phenoxazinium Chlorides Functionalized with Sulfonamide Groups as NIR Fluorescent Probes for Vacuole, Endoplasmic Reticulum, and Plasma Membrane Staining

**DOI:** 10.3390/ijms24033006

**Published:** 2023-02-03

**Authors:** João C. C. Ferreira, Rui P. C. L. Sousa, A. Preto, Maria João Sousa, M. Sameiro T. Gonçalves

**Affiliations:** 1 Centre of Chemistry (CQUM), Department of Chemistry, University of Minho, Campus of Gualtar, 4710-057 Braga, Portugal; 2 Centre of Molecular and Environmental Biology (CBMA), Department of Biology, University of Minho, Campus of Gualtar, 4710-057 Braga, Portugal; 3 Institute of Science and Innovation for Bio-Sustainability (IBS), University of Minho, Campus of Gualtar, 4710-057 Braga, Portugal

**Keywords:** Nile Blue, benzo[*a*]phenoxazines, sulfonamide groups, NIR probes, fluorescent probes

## Abstract

The demand for new fluorophores for different biological target imaging is increasing. Benzo[*a*]phenoxazine derivatives are fluorochromophores that show promising optical properties for bioimaging, namely fluorescent emission at the NIR of the visible region, where biological samples have minimal fluorescence emission. In this study, six new benzo[*a*]phenoxazinium chlorides possessing sulfonamide groups at 5-amino-positions were synthesized and their optical and biological properties were tested. Compared with previous probes evaluated using fluorescence microscopy, using different *S. cerevisiae* strains, these probes, with sulfonamide groups, stained the vacuole membrane and/or the perinuclear membrane of the endoplasmic reticulum with great specificity, with some fluorochromophores capable of even staining the plasma membrane. Thus, the addition of a sulfonamide group to the benzo[*a*]phenoxazinium core increases their specificity and attributes for the fluorescent labeling of cell applications and fractions, highlighting them as quite valid alternatives to commercially available dyes.

## 1. Introduction

The use of fluorescent probes for bioimaging has proved to be an essential and robust tool in the fields of biochemistry, medicine, and environmental sciences [1,2,3]. The proficient characteristics of fluorescent imaging has proved to be fundamental for the real-time monitoring of the localization, behavior, and biological roles of biomolecules in complex living systems [4,5]. In this field, fluorescent probes include synthetic organic dyes that can be used to interact and specifically label a biological target [6,7]. As such, it is not surprising that there is a demand for the development of new fluorophores to overcome the drawbacks of commercial dyes, such as their difficult synthesis, restricted availability, and biological interference [8,9,10]. A fluorescent probe must possess a set of photophysical characteristics that suits it as a robust dye, such as a high molar extinction coefficient, high quantum yield values, minimum photodamage, good stability against photobleaching, as well as high affinity for the specific labelling of biological targets [7,11,12,13]. Ideally, it should absorb and emit fluorescence in the long-wavelength part of the visible range of the spectrum, preferably in the NIR region, as light minimizes damage to biological samples, and decreases the influence of background autofluorescence [14,15,16].

Benzo[*a*]phenoxazinium chlorides are long-wavelength, NIR fluorescent probes that possess exceptional optical properties. They strongly absorb and emit fluorescence up to about 720 nm, with modest Stokes shifts (20–60 nm) and have high photostability, quantum yields, and molar absorption [17,18,19,20,21,22,23,24,25,26,27,28,29,30].

Among these compounds, Nile Blue has been recognized as a lead compound due to its desirable physicochemical and photophysical properties, and has been used as a scaffold for the synthesis of new molecules for fluorescent staining applications [27,31,32].

In addition to their outstanding optical properties, benzo[*a*]phenoxazine compounds have also been reported for their pharmaceutical properties. These compounds were reported for their validity as antitumor agents [33,34], antifungals [23,35,36,37], antibacterials [38,39], antimalarials [40,41], antivirals [42], and as photo-sensitizers in photodynamic therapy [43,44]. Their compact structure, and the presence of amines in 5- and 9-positions allows benzo[*a*]phenoxazinium chlorides to possess different functional groups, resulting in compounds with variable behaviors.

Keeping in mind the physicochemical characteristics, as well as the multiple applications of benzophenoxazinium salts mentioned above, we decided to synthesize a new set of benzophenoxazinium chlorides functionalized with sulfonamide groups. We took this approach because, at least to our knowledge, there are no reports in the literature of the synthesis and characterization of benzo[*a*]phenoxazinium chlorides possessing these functional groups. In fact, sulfonamide or sulfonyl functional groups are of great importance in medicinal chemistry as they exhibit a broad reactivity profile and are considered safe for drug development [45,46,47]. Fluorescent-labelled sulfonamides have also been successfully used to visualize, identify, and monitor intracellular targets [48,49]. In fact, sulfonamides have been reported to behave as a class of electron donors that can be incorporated into both fluorescent and bioluminescent probes [50].

In previous publications, we have reported that the behavior of benzo[*a*]phenoxazinium chlorides depends on the substituents at the 5- and 9-positions of the heterocycle [23,51]. In the present study, we report the synthesis, photophysical, antifungal, and staining characterizations of six new benzo[*a*]phenoxazinium chlorides, functionalized with sulfonamide groups. Our main objective was to evaluate if the introduction of the sulfonamide group into the scaffolds of precursor compounds would be beneficial to the overall biological activity and/or fluorescent labeling capacity.

All the tested compounds exhibited antiproliferative activity against *S. cerevisiae*. Compared with precursor compounds recently reported [23], the addition of the sulfonamide group had a high positive effect on the fluorescence staining pattern. Analysis of the intracellular distribution of the compounds showed that they stain the vacuolar membrane and/or the perinuclear membrane of the endoplasmic reticulum with great specificity, with compounds even capable of staining the plasma membrane, highlighting them as valid molecules to be explored as viable alternatives to the available commercial dyes.

## 2. Results and Discussion

### 2.1. Chemistry

With the aim of obtaining benzo[*a*]phenoxazinium chlorides capable of specifically staining cell organelles, and the purpose of exploring the effects of sulfonamide groups as terminals of 5-amino substituents in combination with different substituents at the 9-amino position of the polycyclic core, a set of new benzo[*a*]phenoxazinium chlorides **3a–f** was designed. 

Different groups were introduced along with the sulfonamide function, namely an alkyl chain (**3a**,**d**,**f**) and a phenyl group with the methyl substituent as an electron donor group (**3b**,**e**) or the nitro group as an electron withdrawing group (**3c**). These variations, along with the differences in the 9-position groups, may allow accessing the most interesting combinations for fluorescence staining.

The synthesis of benzo[*a*]phenoxazinium chlorides **3a–f** started with the synthesis of the precursors **1a–c** and **2a–c**. 5-(Dipropylamino)-2-nitrosophenol hydrochloride **1a**, 2-nitroso-5-(propylamino)phenol hydrochloride **1b**, and 5-(ethylamino)-4-methyl-2-nitrosophenol hydrochloride **1c** were prepared with a reaction of 3-(dipropylamino)phenol, 3-(propylamino)phenol, and 3-(ethylamino)-4-methylphenol, respectively, with sodium nitrite in an acid solution under ice cold conditions [37,52]. *N*^1^-(Naphthalen-1-yl)propane-1,3-diamine hydrobromide was obtained using the alkylation of naphthalen-1-amine with 3-bromopropan-1-amine hydrobromide in ethanol under reflux conditions [52]. Precursors **2a–c** were obtained with a reaction of *N*^1^-(naphthalen-1-yl)propane-1,3-diamine hydrobromide with the corresponding sulfonyl chloride, in dichloromethane, at 0 °C, in the presence of triethylamine. Thus, the reaction of *N*^1^-(naphthalen-1-yl)propane-1,3-diamine hydrobromide with propane-1-sulfonyl chloride, 4-methylbenzene-1-sulfonyl chloride, and 4-nitrobenzene-1-sulfonyl chloride gave *N*-(3-(naphthalen-1-ylamino)propyl)propane-1-sulfonamide **2a**, 4-methyl-*N*-(3-(naphthalen-1-ylamino)propyl)benzenesulfonamide **2b** and *N*-(3-(naphthalen-1-ylamino)propyl)-4-nitrobenzenesulfonamide **2c**. Compound **2a** was isolated by column chromatography as a black oil in a 61% yield. In the case of precursors **2b** and **2c**, the purification was also carried out using column chromatography and gave brown oils, whose ^1^H NMR spectra confirmed the presence of the expected precursors, but together with by-products related to the starting reagents (at about 10 % and 15% in compounds **2b** and **2c**, respectively), and were used in these forms for the following reactions.

**Scheme 1:**
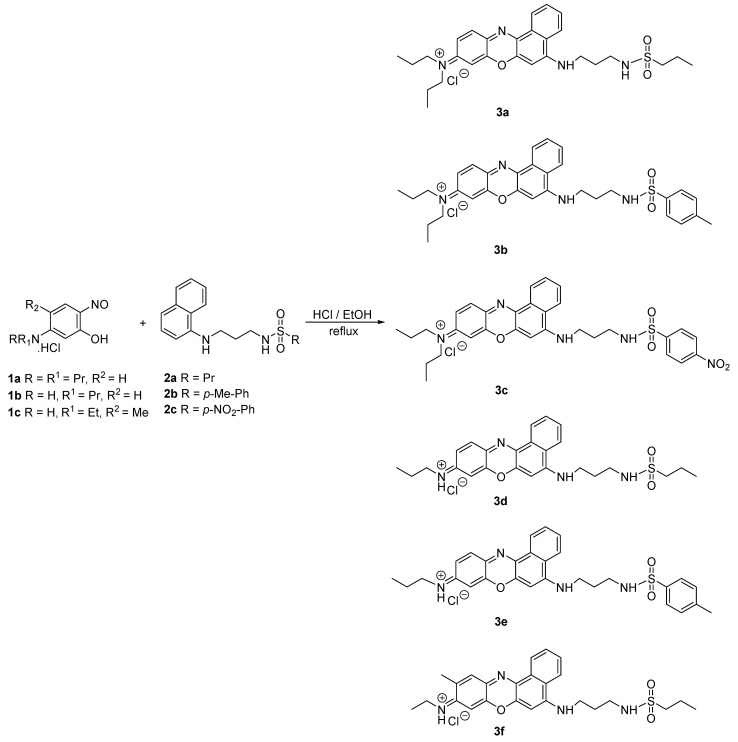
Synthesis of benzo[*a*]phenoxazinium chlorides **3a–f**.

Benzo[*a*]phenoxazinium chlorides **3a–f** were afforded by the reaction between nitrosophenol precursors **1a–c** and naphthylamine derivatives **2a–c**, in acidic medium (Scheme 1). The 9-amino position dialkylated nitrosophenol **1a**, reacted with precursors **2a–c**, at reflux, in methanol, in the presence of concentrated hydrochloric acid, and after silica gel column chromatography purification gave *N*-propyl-*N*-(5-((3-(propylsulfonamido)propyl)amino)-9*H*-benzo[*a*]phenoxazin-9-ylidene)propan-1-aminium chloride **3a**, *N*-(5-((3-(4-methylphenylsulfonamido)propyl)amino)-9*H*-benzo[*a*]phenoxazin-9-ylidene)-*N*-propylpropan-1-aminium chloride **3b** and *N*-(5-((3-(4-nitrophenylsulfonamido)propyl)amino)-9*H*-benzo[*a*]phenoxazin-9-ylidene)-*N*-propylpropan-1-aminium chloride **3c**, respectively. In similar conditions, 5-amino position monoalkylated nitrosophenol **1b** reacted with precursors **2a** and **2b** to yield *N*-(5-((3-(propylsulfonamido)propyl)amino)-9*H*-benzo[*a*]phenoxazin-9-ylidene)propan-1-aminium chloride **3d** and *N*-(5-((3-(4-methylphenylsulfonamido)propyl)amino)-9*H*-benzo[*a*]phenoxazin-9-ylidene)propan-1-aminium chloride **3e**, respectively. At last, nitrosophenol precursor **1c** reacted with naphthylamine precursor **2a** to yield *N*-(10-methyl-5-((3-(propylsulfonamido)propyl)amino)-9*H*-benzo[*a*]phenoxazin-9-ylidene)ethanaminium chloride **3f**. Compounds **3a–f** were obtained as blue solids in yields ranging from 8–48% and were fully characterized using high resolution mass spectrometry and IR and NMR (^1^H and ^13^C) spectroscopy.

FTIR spectra of compounds **3a–f** showed the bands corresponding to amine groups (3437–3170 cm^−1^) and the C-N bond of the central oxazine ring (1641–1638 cm^−1^). Bands corresponding to the S=O bond also appear (1383–1385 and 1159–1187 cm^−1^).

The ^1^H NMR spectra of compounds **3a–f** show the aromatic proton signals in the range *δ* 6.61–8.96 ppm. Compounds **3a–e** show the signal corresponding to H-10 (*δ* 7.11–7.36 ppm); on the other hand, compound **3f** possess a methyl group in the 10-position, that appears in the form of a singlet at *δ* 2.35 ppm. Regarding the 9-position of compounds **3a–e**, the protons of the terminal methyl group appear in the form of a triplet or multiplet (*δ* 1.02–1.12 ppm), the protons of the adjacent methylene group appear in the form of a quintet or multiplet (*δ* 1.74–1.90 ppm), and the protons of the methylene group directly linked to nitrogen appear in the form of a triplet or multiplet (*δ* 3.38–3.67 ppm). Compound **3f** shows the protons of the methylene group directly linked to nitrogen at 3.50–3.60 ppm, in the form of a multiplet, and the terminal CH_3_ group appears as a multiplet (*δ* 1.32–1.39 ppm).

Regarding the 5-position amine substituents, all the compounds possess a propyl group. The protons of the central methylene, NHCH_2_*CH_2_*CH_2_NH, appear in the form of a quintet, multiplet, or extended singlet (*δ* 2.00–2.32 ppm), while the adjacent NHCH_2_CH_2_*CH_2_*NH protons appear in the form of a triplet, multiplet, or extended singlet (*δ* 3.06–3.33 ppm) and NH*CH_2_*CH_2_CH_2_NH appear as an extended triplet, multiplet, or singlet (*δ* 3.74–3.87 ppm). Compounds **3a**, **3d**, and **3f** have the sulfonyl group attached to the propyl group, so their spectra show the signals of the methyl protons in the form of a triplet or multiplet (*δ* 1.02–1.12 ppm), the methylene proton signals NHSO_2_CH_2_*CH_2_*CH_3_ as a sextet or multiplet (*δ* 1.74–1.90 ppm), and NHSO_2_*CH_2_*CH_2_CH_3_ as a triplet or multiplet (*δ* 3.02–3.10 ppm). Spectra of compounds **3b** and **3e** show the signal of the protons of the *para*-methyl group of the aromatic ring attached to the sulfonyl group in the form of a singlet (*δ* 2.36 and 2.38 ppm), as well as by the aromatic protons of the ring (*δ* 7.34 ppm, *ortho*-CH_3_ and *δ* 7.71–7.76 ppm, *meta*-CH_3_). Compound **3c** has the nitro group instead of the methyl group present in the two previous compounds, whose electron withdrawing effect displaces aromatic protons from the *ortho*-position to a higher chemical shift (*δ* 8.33 ppm) relative to the protons at the *meta* position (*δ* 8.08 ppm).

The ^13^C NMR spectra of compounds **3a–f** show the aromatic carbon signals, characteristic of the benzo[*a*]phenoxazine moiety (*δ* 94.01–164.62 ppm). It is possible to observe the differences between compound **3f** and the remaining from the change in the chemical shift of C-10 from 112.02–117.55 ppm to 149.64 ppm.

Regarding the substituents on the 9-position amine, compounds **3a–c** show the signals of the propyl group, namely N(CH_2_CH_2_*CH_3_*)_2_ (*δ* 11.42–11.48 ppm), N(CH_2_*CH_2_*CH_3_)_2_ (*δ* 21.81–21.87 ppm), and N(*CH_2_*CH_2_CH_3_)_2_ (*δ* 54.59–54.64 ppm). These values are slightly different from the values for compounds with only one propyl group as a substituent (**3d**,**e**), which show the signal of the terminal CH_3_ carbon at *δ* 11.79–11.80 ppm, from the central methylene group at *δ* 23.03–23.05 ppm and the CH_2_ group directly linked to the amine at *δ* 46.43–46.44 ppm. The spectrum of compound **3f** shows the methyl group at the 10-position at *δ* 17.61 ppm, and the ethyl group at the 9-position at *δ* 14.12 ppm for CH_3_ and *δ* 39.71 ppm for CH_2_. Regarding the 5-position, the methylene carbons are also highlighted; namely NHCH_2_*CH_2_*CH_2_NHSO_2_ (*δ* 26.56–30.76 ppm), NH*CH_2_*CH_2_CH_2_NHSO_2_ (*δ* 42.28–43.18 ppm), and NHCH_2_CH_2_*CH_2_*NHSO_2_ (*δ* 38.35–41.58 ppm). Compounds **3a**, **3d**, and **3f** show the signals of the carbons of the propyl group linked to the sulfonyl function, namely the methyl group (*δ* 13.21–13.25 ppm) and the methylene carbons, NHSO_2_CH_2_*CH_2_*CH_3_ (*δ* 18.39–18.41 ppm) and NHSO_2_*CH_2_*CH_2_CH_3_ (*δ* 54.44–54.45 ppm). On the other hand, compounds **3b** and **3e** exhibit the signals of the methyl group present in the aromatic ring attached to the sulfonyl function (*δ* 21.40–21.45 ppm) and the signals of aromatic carbons (*δ* 128.07–128.08 ppm, *meta*-CH_3_, and *δ* 130.77–130.80 ppm, *ortho*-CH_3_). In the case of compound **3c**, these signals are also displayed (*δ* 125.45 ppm, *ortho*-CH_3_, and *δ* 129.44 ppm, *meta*-CH_3_).

### 2.2. Photophysical Characterization

Benzo[*a*]phenoxazinium chlorides **3a–f** were studied in different solvents to assess their photophysical properties. The solvents used were dry ethanol, water, and aqueous solutions of different biologically relevant pH values. The maximum absorption (λ_abs_) and emission (λ_emi_) wavelengths (Figures S1–S12), the logarithm of the molar extinction coefficient (log *ε*), the relative fluorescence quantum yield (*Φ*_F_), using Oxazine 1 as a standard (*Φ*_F_ = 0.11 in ethanol) at 590 nm excitation, and the Stokes shifts (Δλ) were obtained for each compound in each solvent. The results obtained are summarized in Table 1.

Photophysical data in ethanol shows that there is a clear difference between di-alkylated compounds at the 9-position (**3a–c**) and mono-alkylated compounds in the same position (**3d**–**f**). A bathochromic shift is seen for compounds with two propyl groups at the 9-position, both in λ_abs_ values (638–641 nm) and in λ_emi_ values (669–671 nm), when compared with mono-alkylated compounds (λ_abs_ 624–631 nm/λ_emi_ 643–646 nm). In addition, a decrease in *Φ*_F_ values is seen for **3a–c** (0.15–0.17) compared with **3d**-**f** (0.33–0.55). Among the latter group, **3e** possesses a different sulfonamide substituent and shows a lower *Φ*_F_ value than the other two compounds. The conjugation of one propyl group at the 9-position and a propyl group as a substituent on the sulfonamide function, at the 5-position, appears to be beneficial for the increase in the fluorescence quantum yield.

Ethanol acidified with trifluoroacetic acid (TFA) was tested to make sure benzophenoxazines were in their acidic forms. As a result, the second absorption band (450–550 nm) showed in dry ethanol spectra for some of the compounds, corresponding to the basic form, disappeared in acidified ethanol. Furthermore, a small increase in both log *ε* and *Φ*_F_ values can be noticed. Compounds **3a**,**c**–**f** show very similar results in this solvent, when compared with ethanol.

In water and aqueous solutions of different pHs, the acid form is less fluorescent and a decrease in *Φ*_F_ values can be seen for all the compounds. A bathochromic shift is observed for all the compounds both in λ_abs_ and λ_emi_ values. At pH 7.4, the basic form of the three compounds appears. Compounds **3d** and **3f** show the most promising fluorescence properties to be applied as NIR bioimaging probes in different biological relevant pHs media.

### 2.3. Biological Activity Evaluation

The biological activity of the six new benzo[*a*]phenoxazinium chlorides **3a–f** was evaluated through a broth microdilution method for testing the antifungal activity against yeasts (M27-A3,CLSI), using *S. cerevisiae* PYCC 4072 as a model organism [53]. The MIC (Minimum Inhibitory Concentration) values that represent the grown of the cultures are listed in Table 2. Lipophilicity of a molecule is an important physical parameter when considering biological activity as it is directly related to the ability of a compound to enter the cell and diffuse intracellularly. This property can be evaluated with the logarithm of its partition coefficient between *n*-octanol and water, i.e., log (c_octanol_/c_water_) or log *P* [44]. Thus, the determination of the partition coefficient (log *P*) [54] was performed using online software *ds* and the results are shown in Table 2 [55]. Compounds with higher log *P* values have low affinity for an aqueous environment and a high affinity for membranous systems.

As can be seen in Table 2, all compounds tested showed antiproliferative activity against the yeast *S. cerevisiae*, with MIC values ranging from 12.5 to >100 µM (compound **3e** showed antifungal activity at 100 µM, but due to its limited solubility, we were unable to increase the concentration to achieve 80% inhibition of cell growth). Comparing these results with those of structurally similar benzo[*a*]phenoxazines, without the sulfonamide group [23], it can be noticed that the introduction of this functional group does not result in an improvement trend in the biological activity of the compounds.

Among the six tested compounds, the di-alkylated derivatives (**3a–c**) show the best biological activity; the most active, compound **3b**, displays a MIC value inferior to the corresponding analogue without the sulfonamide group (MIC 12.5 μM instead of 25 μM) [23]. The conjugation of these results with the optical properties of the set shows that compounds **3d**–**f** may be the most promising benzo[*a*]phenoxazines for the fluorescent staining of biological samples as they have low toxicity.

### 2.4. Fluorescence Microscopy Studies

#### 2.4.1. General Observations

**Figure 1:**
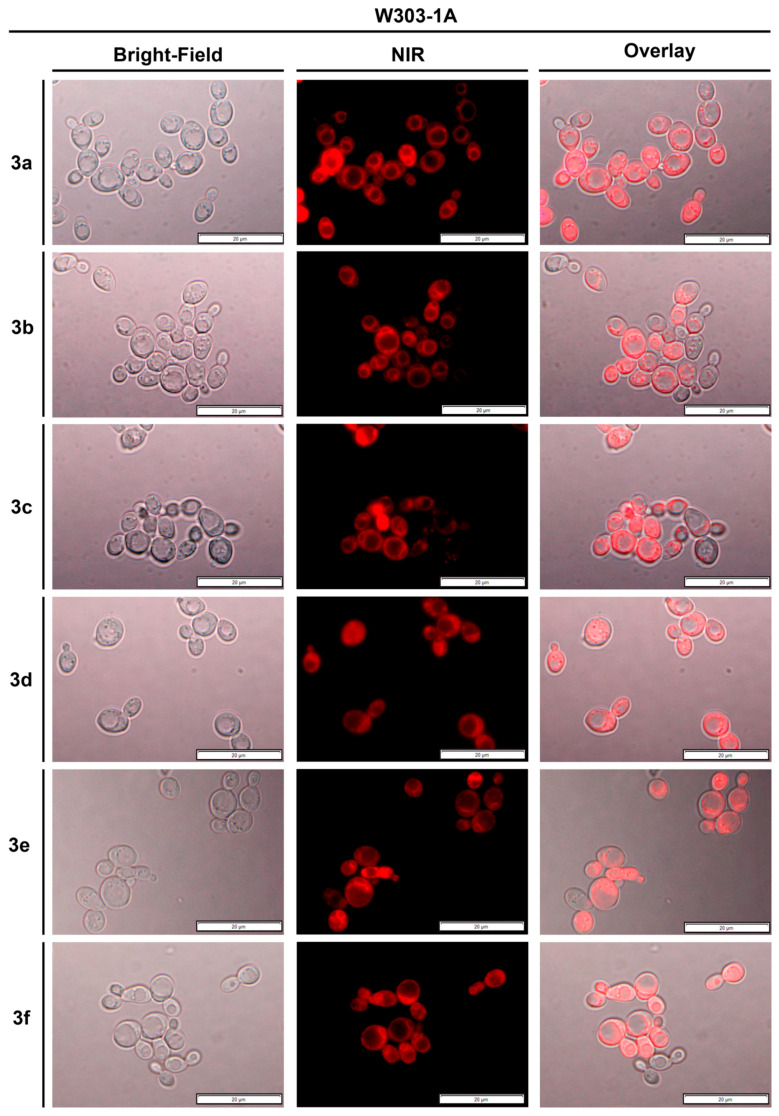
**Intracellular distribution of benzo[*a*]phenoxazinium chlorides 3a–f.** Fluorescence microscopy images of W303-1A cells after incubation with **3a–f** (MIC Concentrations) for 1 h. Samples were rinsed two times in PBS 1× and mounted on a slide for observation with a 60× oil immersion objective, using the adequate filters U-FDICT, ET-CY5-FAR-RED (exciter filter: 595–645 nm/emission filter: 665–715 nm). Representative images of three independent experiments. Scale bar 20 µm.

To evaluate the fluorescent staining capacity of the compounds, the intracellular distribution of the benzo[*a*]phenoxazinium chlorides **3a–f** was assessed using fluorescence microscopy. To obtain preliminary information on the staining ability of these compounds, MIC values were used as reference concentrations. *S. cerevisiae* W303-1A cells were incubated with MIC concentrations for 1 h and observed under the fluorescence microscope. This incubation resulted in the accumulation of the compounds in the cells, as NIR fluorescence emission was observed for all compounds upon excitation (Figure 1). Looking closely at Figure 1, it can be observed that these compounds appear to accumulate particularly at the vacuolar membrane and/or at the perinuclear membrane of the endoplasmic reticulum, which is consistent with our previous observations for this class of compounds [23,35,36]. In fact, Compounds **3a** and **3b** have a high specific labeling for the vacuole membrane and perinuclear membrane of the endoplasmic reticulum. Compound **3c** shows an identical labeling pattern, but with lower specificity. A more unspecific labeling pattern is observed for compounds **3d**–**f**, probably due to the high concentration used for these compounds. However, the tendency to accumulate in the endoplasmic reticulum and in some cells in the vacuolar membrane is observed.

Furthermore, these preliminary observations allowed us to conclude that the addition of the sulfonamide group significantly improves the specificity of labeling compared with compounds of a similar structure that lack this functional group [23].

#### 2.4.2. Vacuole, Perinuclear Membrane of the Endoplasmic Reticulum and Plasma Membrane Stain

To confirm our previous observations and to evaluate the fluorescence pattern of each compound individually, we analyzed the specific intracellular distribution of the compounds using *S. cerevisiae* cells expressing GFP-tagged proteins located at the vacuole (W303-1A Vba1-GFP—expressing a GFP-tagged form of the vacuole membrane transporter Vba1 [56]), at the endoplasmic reticulum (BY4741 Sec66-GFP—expressing a GFP-tagged non-essential subunit of Sec63 ER complex [57]) and at the plasma membrane (23344c *jen1*Δ Jen1-GFP—expressing a GFP-tagged functional lactate transporter Jen1 [58]). We also reduced the concentration of the compounds and stained the cells with 2.5 µM of **3a–f**, as we had previously observed that this concentration increased the specificity and labeling ability of the compounds of this family [35].

**Figure 2:**
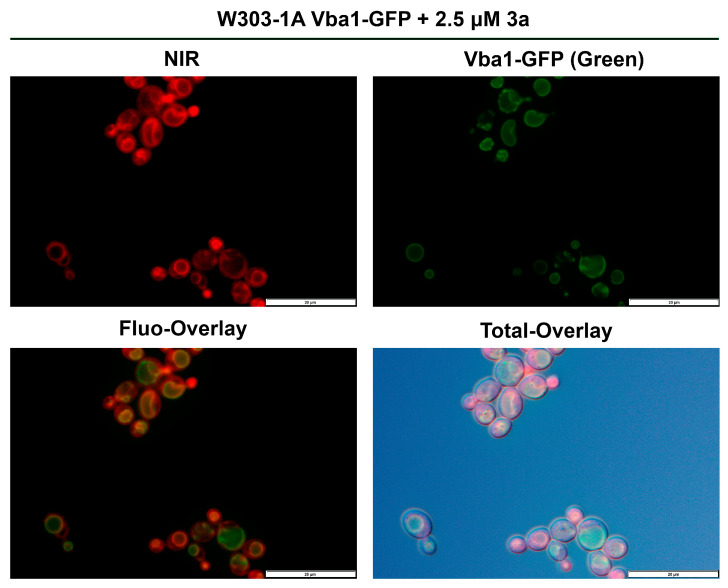

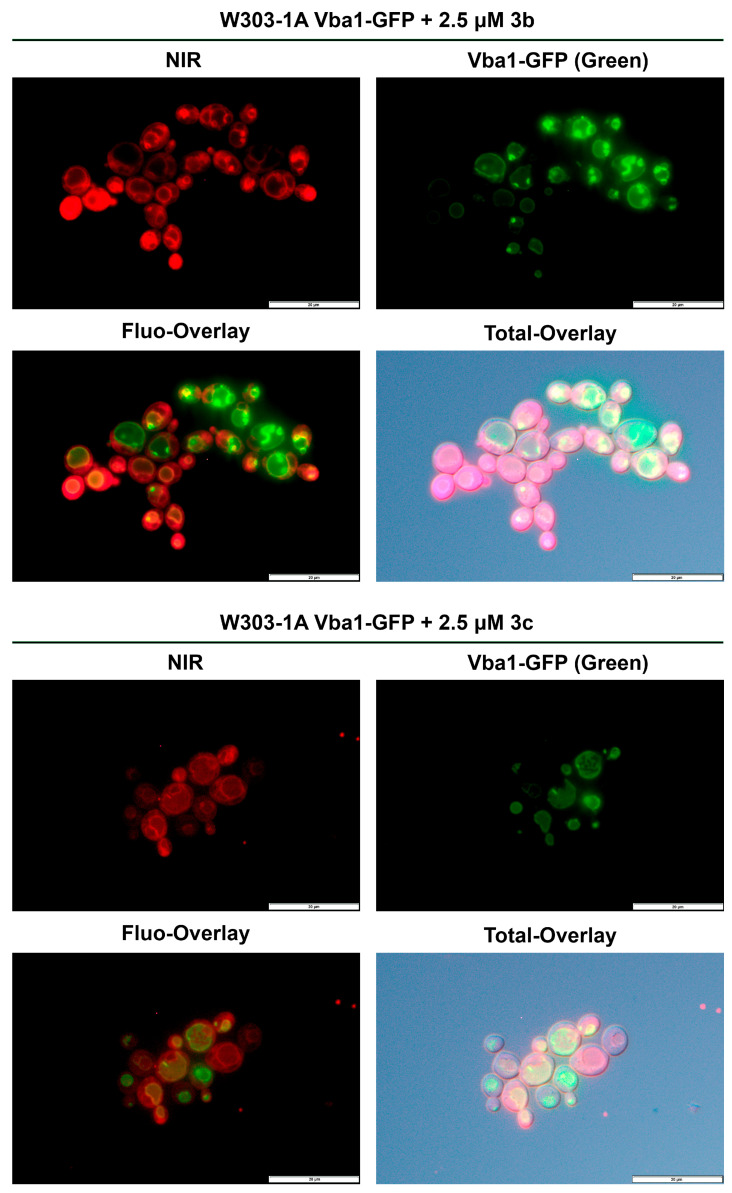

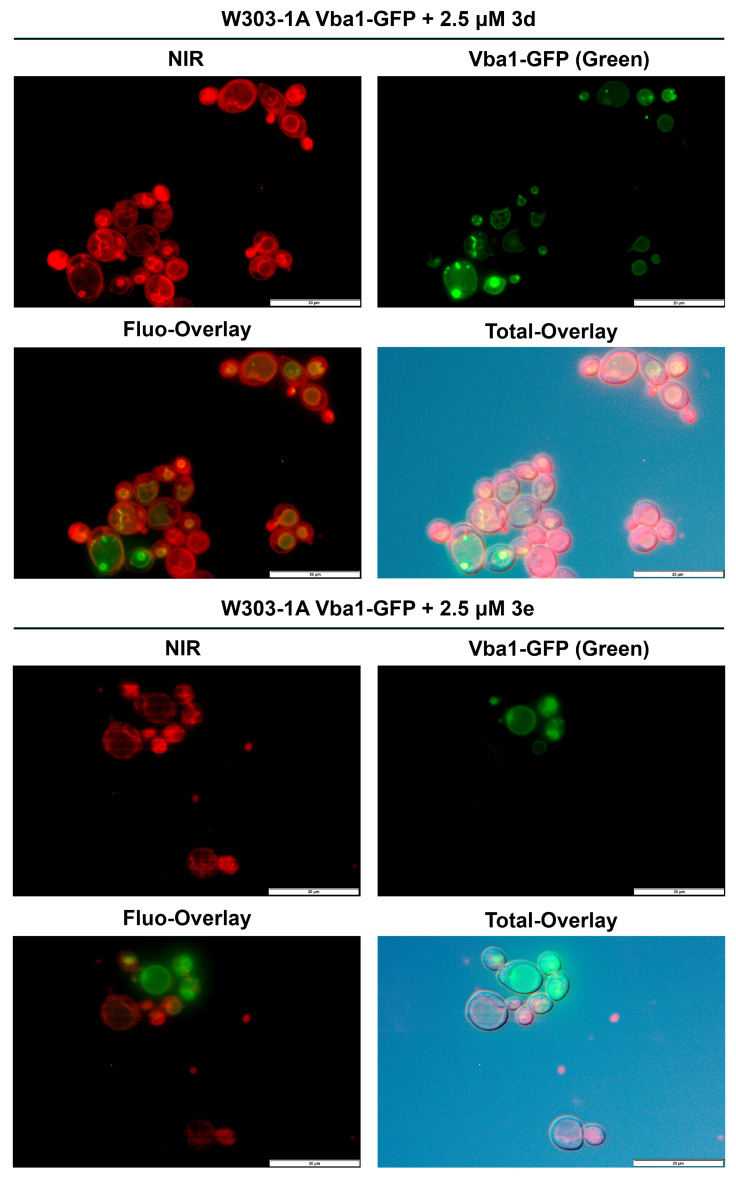

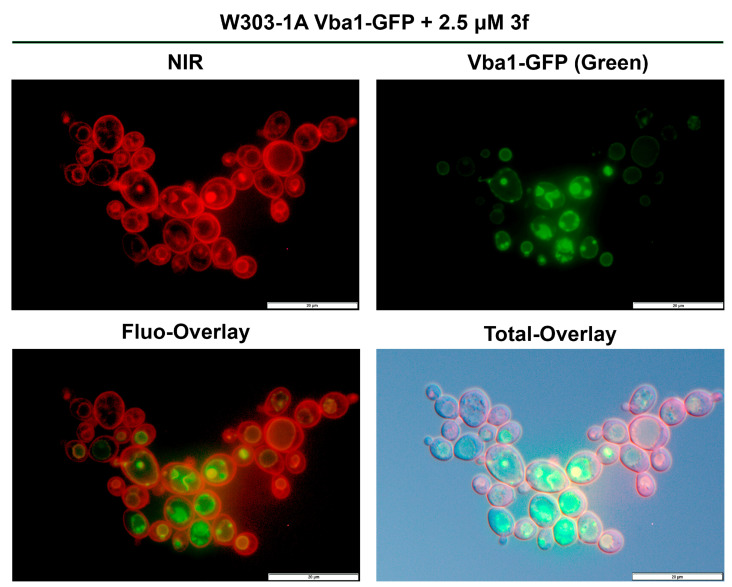
**Co-localization of benzo[*a*]phenoxazinium chlorides 3a–f with vacuolar membrane.** Fluorescence microscopy images of W303-1A Vba1-GFP cells after incubation with **3a–f** (2.5 µM) for 1 h. Samples were rinsed two times in PBS 1× and mounted on a slide for observation with a 60× oil immersion objective, using the adequate filters U-FDICT, ET-CY5-FAR-RED (exciter filter: 595–645 nm/emission filter: 665–715 nm), and TLV-U-FF-FITC (exciter filter: 455–495 nm/emission filter: 505–555 nm). Representative images of three independent experiments. Scale bar 20 µm.

**Figure 3:**
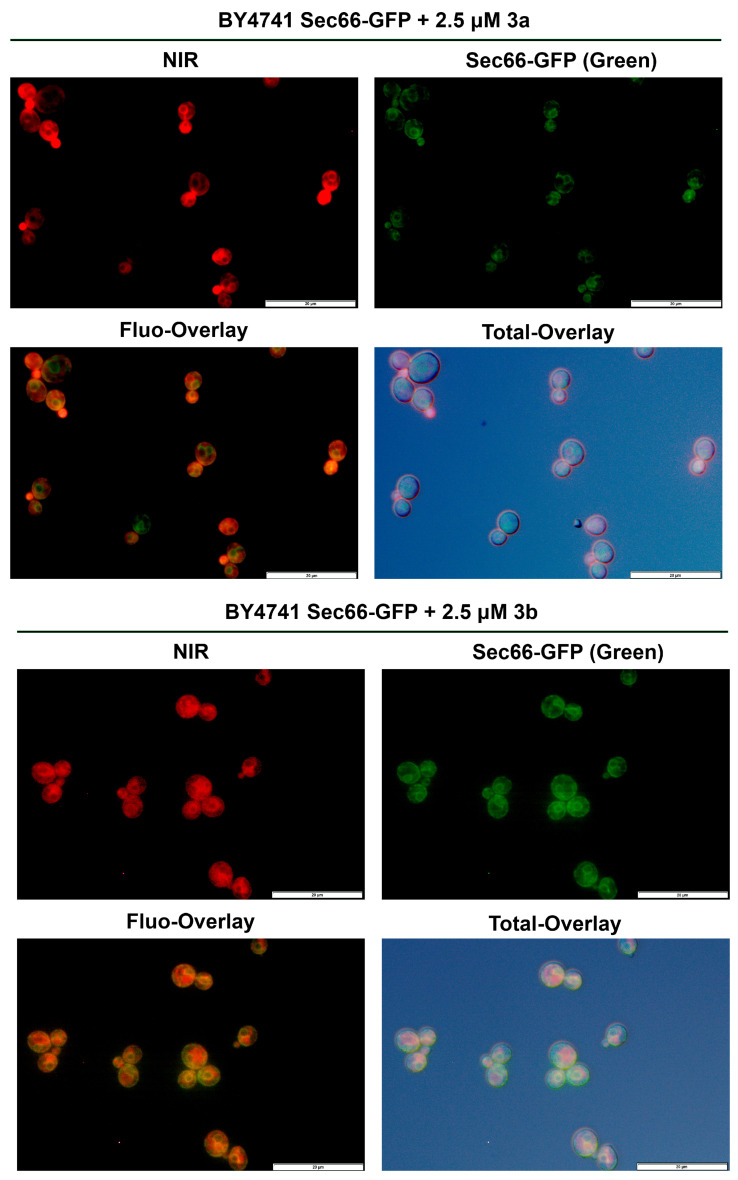

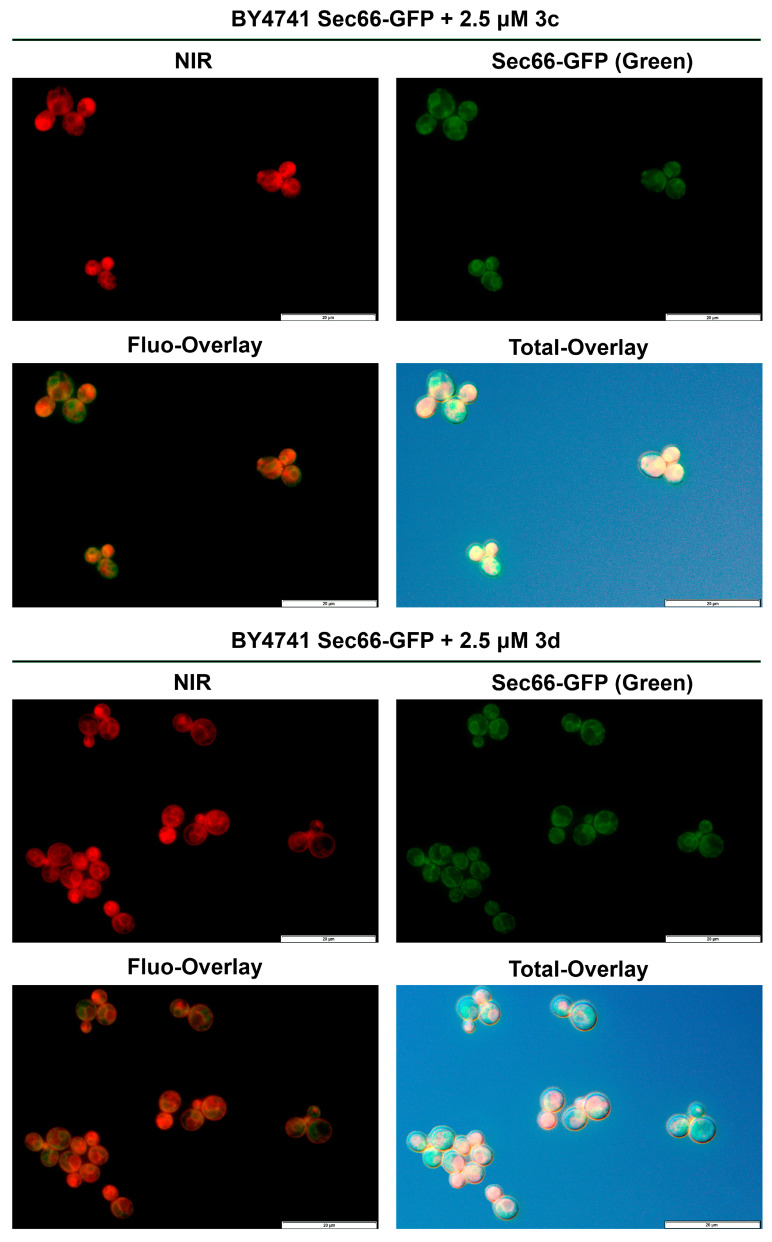

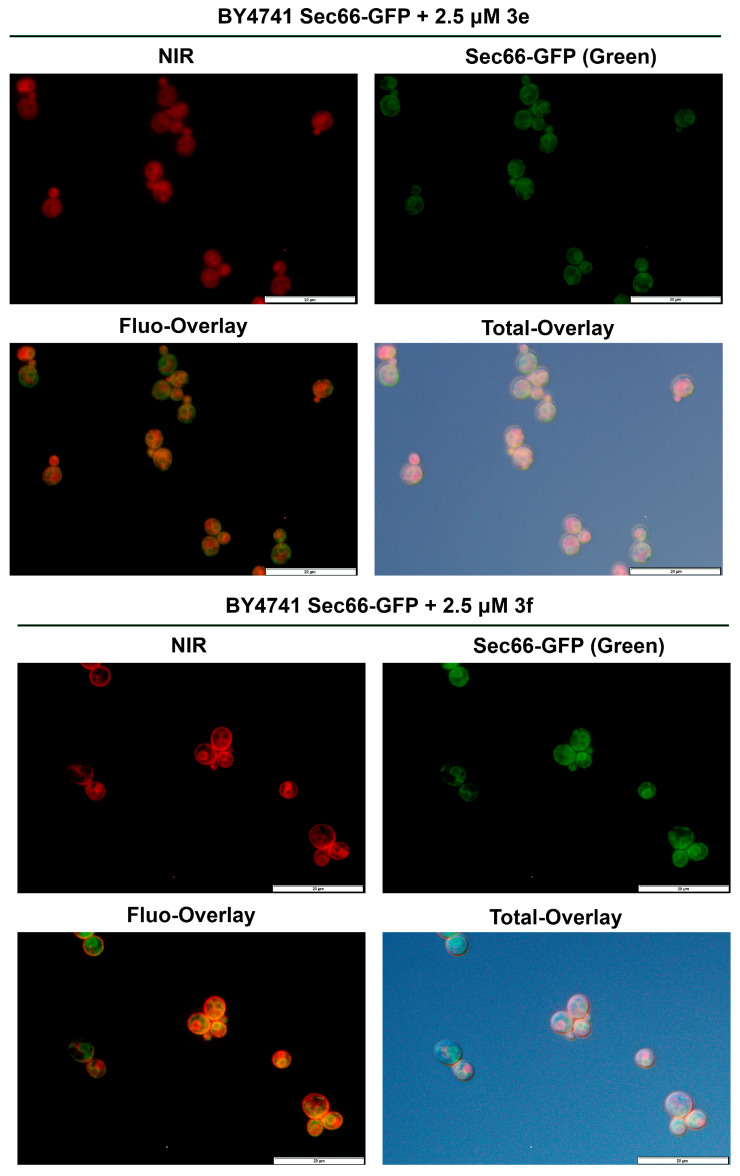
**Co-localization of benzo[*a*]phenoxazinium chlorides 3a–f with perinuclear membrane of the endoplasmic reticulum.** Fluorescence microscopy images of BY4741 Sec66-GFP cells after incubation with **3a–f** (2.5 µM) for 1 h. Samples were rinsed two times in PBS 1× and mounted on a slide for observation with a 60× oil immersion objective, using the adequate filters U-FDICT, ET-CY5-FAR-RED (exciter filter: 595–645 nm/emission filter: 665–715 nm), and TLV-U-FF-FITC (exciter filter: 455–495 nm/emission filter: 505–555 nm). Representative images of three independent experiments. Scale bar 20 µm.

**Figure 4:**
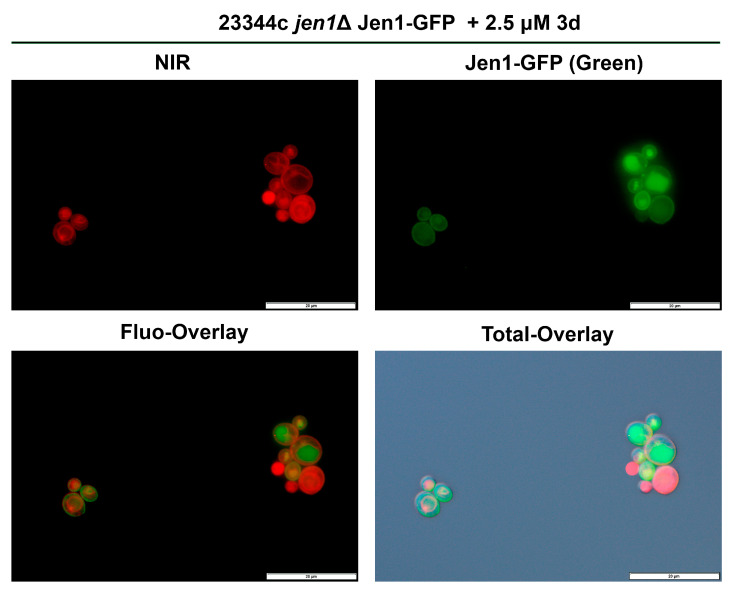

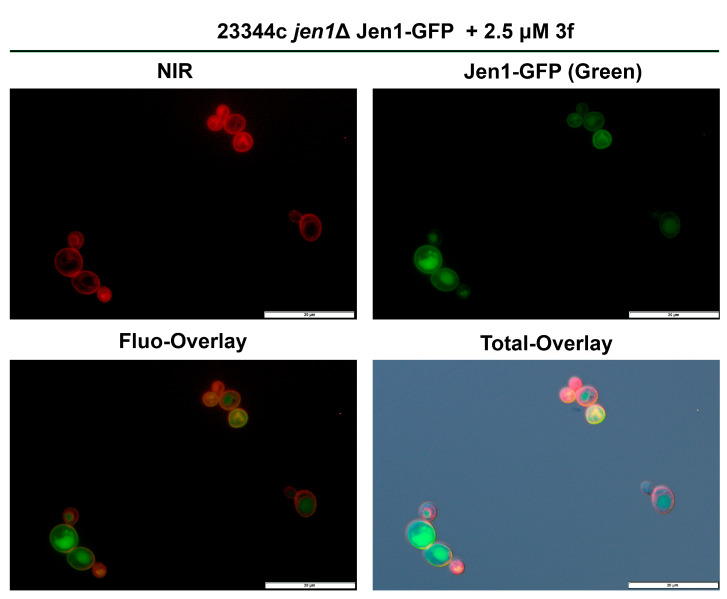
**Co-localization of benzo[*a*]phenoxazinium chlorides 3d and 3f with plasma membrane.** Fluorescence microscopy images of 23344c *jen1*Δ Jen1-GFP cells after incubation with **3d** and **3f** (2.5 µM) for 1 h. Samples were rinsed two times in PBS 1× and mounted on a slide for observation with a 60× oil immersion objective, using the adequate filters U-FDICT, ET-CY5-FAR-RED (exciter filter: 595–645 nm/emission filter: 665–715 nm), and TLV-U-FF-FITC (exciter filter: 455–495 nm/emission filter: 505–555 nm). Representative images of three independent experiments. Scale bar 20 µm.

As previously reported, reducing the concentration of the compounds proved to be beneficial for live-cell imaging experiments. More specific staining patterns and clearer phenotypes were obtained. Staining experiments with compound **3a** demonstrated the specificity of this compound to accumulate and stain the vacuolar membrane, as seen from the co-localization of NIR fluorescence and green fluorescence of Vba1-GFP (Figure 2 and Figure 3). In addition, perinuclear ER membrane labeling can also be observed in some cells. Compounds **3b**, **3c**, and **3e** showed the ability to accumulate and stain both the vacuolar membrane and the perinuclear ER membrane, as shown by the co-localization between the NIR and green fluorescence in both strains W303-1A Vba1-GFP and BY4741 Sec66-GFP (Figure 2 and Figure 3). However, the quality and resolution of the staining of compounds **3c** and **3e** were not as good as the other compounds. Compounds **3d** and **3f**, which have the propyl sulfonamide group, are those with the highest relative fluorescence quantum yields (Table 1). Consistent with this is their excellent performance in fluorescence staining. These compounds, which show strong structural similarities, being mono-alkylated in the 9-position, besides possessing the same substituent group (propyl sulfonamide), showed a very clear labeling pattern with specificity for the vacuole and the perinuclear ER membrane (Figure 2 and Figure 3). Moreover, at a concentration of 2.5 µM, there was an accumulation of the compounds at the plasma membrane. This was evidenced with co-localization with the Jen1 transporter, which is mainly localized in the plasma membrane after expression induction (Figure 4). In some cells, it is also possible to observe Jen1 inside the vacuole, as this phenotype was also reported for this transporter [58].

Overall, the compounds synthesized were found to show high resolution fluorescence staining patterns demonstrating the ability to strain relevant intracellular structures. Indeed, compounds **3d** and **3f** were the ones that exhibited the sharpest staining phenotypes and better photophysical values of the series. However, compared with their precursors, they did not show significant improvement in their photophysical properties [23]. Compound **3f** even exhibited lower fluorescence quantum yields than its precursor, which in a way shows that the sharpest fluorescence staining phenotypes of these compounds do not depend only on their photophysical properties. In our view, one of the reasons for the better fluorescence staining patterns of the sulfonamide-functionalized compounds seems to be correlated to their higher lipophilicity (indicated by their higher partition coefficient (log *P*) values—Table 2), which is theoretically correlated to their higher accumulation at the intramembrane regions and thus subsequently leads to a sharper phenotype of the staining of these structures.

In fact, the compounds containing sulfonamide groups have proven to be the ones that exhibit the best fluorescence labeling patterns of our entire library. Moreover, the fact that these compounds have high MICs and retain their ability to stain cellular structures at concentrations well below their MICs is a positive aspect for their use as fluorescent probes, since it is desirable that a probe does not affect the viability of a given organism.

## 3. Materials and Methods

### 3.1. Synthesis General

Melting points were correct and were measured on a Stuart SMP3 melting point apparatus. TLC analyses were carried out on 0.20 mm thick precoated silica plates (Macherey-Nagel, Dueren, Germany), and spots were visualized under UV light on a CN-6 camera. Chromatography on silica gel was carried out on Acros Organics 60 (0.035–0.070 mm) (Geel, Belgium). IR spectra were determined on a BOMEM MB 104 spectrophotometer, Quebec City, QC, Canada. Samples were prepared in 1% KBr pellets. UV–Vis–NIR absorption spectra (200–800 nm) were obtained using Shimadzu UV/3101PC, Kyoto, Japan, spectrophotometer and fluorescence spectra with Fluoromax-4 spectrofluorometer, Horiba, NJ, USA. NMR spectra were obtained on a Bruker Avance III 400 at an operating frequency of 400 MHz for ^1^H and 100 MHz for ^13^C using the solvent peak as internal reference at 25 °C, Billerica, MA, USA. All chemical shifts are given in ppm using *δ*_H_ Me_4_Si = 0 ppm as reference and *J* values are given in Hz. Assignments were made through a comparison of chemical shifts, peak multiplicities, and *J* values, and were supported spin decoupling-double resonance and bidimensional heteronuclear correlation techniques. Mass spectrometry analyses were performed at the “C.A.C.T.I.—Unidad de Espectrometria de Masas”, at University of Vigo, Pontevedra, Spain. All commercial reagents and solvents were used as received.

### 3.2. General Procedure for the Synthesis of N-Substituted Naphthylamine Derivatives ***2a–c***

To a stirring solution of *N*^1^-(naphthalen-1-yl)propane-1,3-diamine hydrobromide [59] in dichloromethane (3 mL), at 0 °C, triethylamine was added followed by the corresponding sulfonyl chloride. The progress of the reaction was monitored with TLC (mixtures of dichloromethane/methanol). After evaporation of the solvent and column chromatography purification on silica gel (mixtures of increasing polarity of dichloromethane/methanol as the eluent), the corresponding *N*-substituted naphthylamine derivative **2a**–**c** was obtained.

#### 3.2.1. *N*^1^-(3-(Naphthalen-1-ylamino)propyl)propane-1-sulfonamide **2a**

*N*^1^-(Naphthalen-1-yl)propane-1,3-diamine hydrobromide (0.250 g, 8.90 × 10^−4^ mol, 1 eq.), triethylamine (0.252 mL, 1.96 × 10^−3^ mol, 2.2 eq.), and propane-1-sulfonyl chloride (0.110 mL, 9.80 × 10^−4^ mol, 1.1 eq.) were stirred for 7h. Triethylamine (0.126 mL, 9.80 × 10^−4^ mol, 1.1 eq.) and propane-1-sulfonyl chloride (0.055 mL, 4.90 × 10^−4^ mol, 0.55 eq.) were once again added and the reaction mixture was stirred for more 7 h. Compound **2a** was obtained as a black oil (0.167 g, 61%). R*f* = 0.85 (dichloromethane/methanol 9:1). ^1^H NMR *δ*_H_ (CDCl_3_, 400 MHz) 1.03 (t, *J* = 5.6 Hz, 3H, NHSO_2_CH_2_CH_2_*CH_3_*), 1.77–1.87 (m, 2H, NHSO_2_CH_2_*CH_2_*CH_3_), 2.00 (quint, *J* = 6.4 Hz, 2H, NHCH_2_*CH_2_*CH_2_NHSO_2_), 2.94–3.04 (m, 2H, NHSO_2_*CH_2_*CH_2_CH_3_), 3.25–3.33 (m, 2H, NHCH_2_CH_2_*CH_2_*NHSO_2_), 3.46 (t, *J* = 6.4 Hz, 2H, NH*CH_2_*CH_2_CH_2_NHSO_2_), 4.76 (broad s, 1H, NHCH_2_CH_2_CH_2_*NH*SO_2_), 6.65 (d, *J* = 7.2 Hz, 1H, H-2), 7.26 (d, *J* = 7.2 Hz, 1H, H-4), 7.35 (t, *J* = 7.6 Hz, 1H, H-3), 7.43–7.48 (m, 2H, H-6 and H-7), 7.78–7.81 (m, 1H, H-8), 7.84–7.89 (m, 1H, H-5) ppm. ^13^C NMR *δ*_C_ (CDCl_3_, 100 MHz) 12.88 (NHSO_2_CH_2_CH_2_*CH_3_*), 17.36 (NHSO_2_CH_2_*CH_2_*CH_3_), 29.31 (NHCH_2_*CH_2_*CH_2_NHSO_2_), 41.11 (NH*CH_2_*CH_2_CH_2_NHSO_2_ and NHCH_2_CH_2_*CH_2_*NHSO_2_), 54.39 (NHSO_2_*CH_2_*CH_2_CH_3_), 104.75 (C-2), 117.85 (C-4), 119.98 (C-5), 123.61 (C-8a), 124.93 (C-6), 125.82 (C-7), 126.44 (C-3), 128.61 (C-8), 134.32 (C-4a), 142.72 (C-1) ppm.

#### 3.2.2. 4-Methyl-*N*-(3-(naphthalen-1-ylamino)propyl)benzenesulfonamide **2b**

The reaction between *N*^1^-(naphthalen-1-yl)propane-1,3-diamine hydrobromide (0.100 g, 3.56 × 10^−4^ mol, 1 eq.), triethylamine (0.100 mL, 7.83 × 10^−4^ mol, 2.2 eq.), and 4-methylbenzene-1-sulfonyl chloride (0.056 g, 3.92 × 10^−4^ mol, 1.1 eq.) (stirring time: 9 h) gave a brown oil (0.055 g), whose ^1^H NMR spectrum showed to be compound **2b** in a purity percentage of approximately 90% (with by-products related to the starting materials), R*f* = 0.73 (dichloromethane/methanol 95:5). ^1^H NMR *δ*_H_ (DMSO-*d_6_*, 400 MHz) 1.77 (quint, *J* = 6.8 Hz, 2H, NHCH_2_*CH_2_*CH_2_NHSO_2_), 2.34 (s, 3H, SO_2_C_6_H_4_*CH_3_*), 2.87 (q, *J* = 6.8 Hz, 2H, NHCH_2_CH_2_*CH_2_*NHSO_2_), 3.16 (q, *J* = 7.2 Hz, 2H, NH*CH_2_*CH_2_CH_2_NHSO_2_), 6.01 (t, *J* = 5.6 Hz, 1H, NH), 6.40 (d, *J* = 8.0 Hz, 1H, H-2), 7.07 (d, *J* = 8.0 Hz, 1H, H-4), 7.24 (t, *J* = 8.0 Hz, 1H, H-3), 7.30–7.43 (m, 4H, H-6, H-7 and 2 × Ar-H *o-*CH_3_), 7.65 (d, *J* = 8.0 Hz, 2H, 2 × Ar-H *m*-CH_3_), 7.72 (d, *J* = 8.0 Hz, 1H, H-8), 8.07 (d, *J* = 8.4 Hz, 1H, H-5) ppm.

#### 3.2.3. *N*-(3-(Naphthalen-1-ylamino)propyl)-4-nitrobenzenesulfonamide **2c**

The reaction between *N*^1^-(naphthalen-1-yl)propane-1,3-diamine hydrobromide (0.100 g, 3.56 × 10^−4^ mol, 1 eq.), triethylamine (0.100 mL, 7.83 × 10^−4^ mol, 2.2 eq.), and 4-nitrobenzene-1-sulfonyl chloride (0.087 g, 3.92 × 10^−4^ mol, 1.1 eq.) (stirring time: 5 h) gave compound **2c** as a brown oil (0.063 g), whose ^1^H NMR spectrum showed to be compound **2c** in a purity percentage of approximately 85% (with by-products related to the starting materials), R*f* = 0.79 (dichloromethane/methanol 95:5). ^1^H NMR *δ*_H_ (DMSO-*d_6_*, 400 MHz) **2c** identified signals: 1.75–1.82 (m, 2H, NHCH_2_*CH_2_*CH_2_NHSO_2_), 2.95–3.01 (m, 2H, NHCH_2_CH_2_*CH_2_*NHSO_2_), 3.10–3.20 (m, 2H, NH*CH_2_*CH_2_CH_2_NHSO_2_), 6.00 (t, *J* = 5.6 Hz, 1H, NH), 6.37 (d, *J* = 7.6 Hz, 1H, H-2), 7.05 (d, *J* = 8.0 Hz, 1H, H-4), 7.21 (t, *J* = 8.0 Hz, 1H, H-3) ppm.

### 3.3. General Procedure for the Synthesis of Benzo[a]phenoxazinium Chlorides ***3a–f***

To a solution of the corresponding nitrosophenol hydrochloride **1a–c** in methanol (3 mL), concentrated hydrochloric acid was added followed by *N*-substituted naphthylamine derivative (**2a–c**), and the resulting solution was refluxed. The progress of the reaction was monitored with TLC (mixtures of dichloromethane/methanol). After evaporation of the solvent and column chromatography purification on silica gel (mixtures of increasing polarity of dichloromethane/methanol as the eluent), the corresponding benzo[*a*]phenoxazinium chloride **3a–f** was obtained.

#### 3.3.1. *N*-Propyl-*N*-(5-((3-(propylsulfonamido)propyl)amino)-9*H*-benzo[*a*]phenoxazin-9-ylidene)propan-1-aminium Chloride **3a**

The reaction between 5-(dipropylamino)-2-nitrosophenol hydrochloride **1a** [37] (0.191 g, 8.59 × 10^−4^ mol, 2 eq.), concentrated hydrochloric acid (0.232 mL), and **2a** (0.131 g, 4.29 × 10^−4^ mol, 1 eq.) (reflux time: 21 h) gave compound **3a** as a blue solid (0.078 g, 33%). Mp = 214.5–216.1 °C. R*f* = 0.48 (dichloromethane/methanol 95:5). FTIR (KBr 1%): *υ*_max_ 3417, 3170, 2958, 2927, 2869, 1638, 1584, 1546, 1493, 1451, 1432, 1383, 1332, 1291, 1237, 1209, 1163, 1131, 1102, 1056, 1010, 820 cm^−1^. ^1^H NMR *δ*_H_ (CD_3_OD, 400 MHz) 1.02–1.12 (m, 9H, N(CH_2_CH_2_*CH_3_*)_2_ and NHSO_2_CH_2_CH_2_*CH_3_*), 1.75–1.87 (m, 6H, N(CH_2_*CH_2_*CH_3_)_2_ and NHSO_2_CH_2_*CH_2_*CH_3_), 2.07–2.17 (m, 2H, NHCH_2_*CH_2_*CH_2_NHSO_2_), 3.09 (t, *J* = 7.6 Hz, 2H, NHSO_2_*CH_2_*CH_2_CH_3_), 3.25–3.33 (m, 2H, NHCH_2_CH_2_*CH_2_*NHSO_2_), 3.63 (t, *J* = 8.0 Hz, 4H, N(*CH_2_*CH_2_CH_3_)_2_), 3.85 (t, *J* = 6.4 Hz, 2H, NH*CH_2_*CH_2_CH_2_NHSO_2_), 6.90 (s, 1H, H-8), 7.00 (s, 1H, H-6), 7.30 (dd, *J* = 9.6 and 2.0 Hz, 1H, H-10), 7.83 (t, *J* = 7.2 Hz, 1H, H-3), 7.88 (d, *J* = 9.2 Hz, 1H, H-11), 7.94 (t, *J* = 8.0 Hz, 1H, H-2), 8.33 (d, *J* = 8.4 Hz, 1H, H-4), 8.92 (d, *J* = 8.0 Hz, 1H, H-1) ppm. ^13^C NMR *δ*_C_ (CD_3_OD, 100 MHz) 11.44 (N(CH_2_CH_2_*CH_3_*)_2_), 13.25 (NHSO_2_CH_2_CH_2_*CH_3_*), 18.41 (NHSO_2_CH_2_*CH_2_*CH_3_), 21.81 (N(CH_2_*CH_2_*CH_3_)_2_), 30.24 (NHCH_2_*CH_2_*CH_2_NHSO_2_), 41.33 (NHCH_2_CH_2_*CH_2_*NHSO_2_), 42.99 (NH*CH_2_*CH_2_CH_2_NHSO_2_), 54.45 (NHSO_2_*CH_2_*CH_2_CH_3_), 54.59 (N(*CH_2_*CH_2_CH_3_)_2_), 94.48 (C-6), 97.19 (C-8), 117.04 (C-10), 123.83 (C-4), 124.75 (Ar-C), 125.63 (C-1), 130.94 (C-3), 131.90 (Ar-C), 132.65 (Ar-C), 132.99 (C-2), 134.10 (C-11), 134.99 (Ar-C), 149.72 (Ar-C), 153.30 (Ar-C), 156.22 (C-9), 159.34 (C-5) ppm. HRMS: m/z (ESI): Found [M+1]^+^: 509.2580; C_28_H_37_N_4_O_3_S requires [M+1]^+^: 509.2581.

#### 3.3.2. *N*-(5-((3-(4-Methylphenylsulfonamido)propyl)amino)-9*H*-benzo[*a*]phenoxazin-9-ylidene)-*N*-propylpropan-1-aminium Chloride **3b**

The reaction between 5-(dipropylamino)-2-nitrosophenol hydrochloride **1a** (0.080 g, 3.10 × 10^−4^ mol, 2 eq.), concentrated hydrochloric acid (0.070 mL), and **2b** (0.055 g, 1.55 × 10^−4^ mol, 1 eq.) (reflux time: 14 h) gave compound **3b** as a blue solid (0.037 g, 40%). Mp > 300 °C. R*f* = 0.52 (dichloromethane/methanol 95:5). FTIR (KBr 1%): *υ*_max_ 3436, 3207, 3062, 2963, 2931, 2874, 1641, 1587, 1547, 1497, 1452, 1437, 1384, 1332, 1290, 1240, 1159, 1135, 1097, 1008, 964, 918, 869, 816 cm^−1^. ^1^H NMR *δ*_H_ (CD_3_OD, 400 MHz) 1.08 (t, *J* = 7.2 Hz, 6H, N(CH_2_CH_2_*CH_3_*)_2_), 1.80 (sext, *J* = 7.2 Hz, 4H, N(CH_2_*CH_2_*CH_3_)_2_), 2.05 (broad s, 2H, NHCH_2_*CH_2_*CH_2_NHSO_2_), 2.38 (s, 3H, Ph-*CH_3_*), 3.06 (broad s, 2H, NHCH_2_CH_2_*CH_2_*NHSO_2_), 3.60 (t, *J* = 8.0 Hz, 4H, N(*CH_2_*CH_2_CH_3_)_2_), 3.74 (broad s, 2H, NH*CH_2_*CH_2_CH_2_NHSO_2_), 6.77 (d, *J* = 2.4 Hz, 1H, H-8), 6.79 (s, 1H, H-6), 7.23 (dd, *J* = 9.6 and 2.4 Hz, 1H, H-10), 7.34 (d, *J* = 8.0 Hz, 2H, 2 × Ar-H *o*-CH_3_), 7.71–7.76 (m, 4H, H-3, H-11 and 2 × Ar-H *m*-CH_3_), 7.84 (t, *J* = 7.6 Hz, 1H, H-2), 8.20 (broad s, 1H, H-4), 8.69 (d, *J* = 8.0 Hz, 1H, H-1) ppm. ^13^C NMR *δ*_C_ (CD_3_OD, 100 MHz) 11.48 (N(CH_2_CH_2_*CH_3_*)_2_), 21.43 (Ph-*CH_3_*), 21.87 (N(CH_2_*CH_2_*CH_3_)_2_), 29.56 (NHCH_2_*CH_2_*CH_2_NHSO_2_), 41.58 (NHCH_2_CH_2_*CH_2_*NHSO_2_), 43.18 (NH*CH_2_*CH_2_CH_2_NHSO_2_), 54.64 (N(*CH_2_*CH_2_CH_3_)_2_), 94.42 (C-6), 97.17 (C-8), 117.04 (C-10), 123.81 (C-4), 124.55 (Ar-C), 125.43 (C-1), 128.08 (2 × Ar-H *m*-CH_3_), 130.80 (2 × Ar-H *o*-CH_3_), 130.84 (C-3), 131.69 (Ar-C), 132.26 (Ar-C), 132.90 (C-2), 134.01 (C-11), 134.59 (Ar-C), 138.45 (*C*-SO_2_), 144.83 (*C*-CH_3_), 149.44 (Ar-C), 152.79 (Ar-C), 156.13 (C-9), 158.99 (C-5) ppm. HRMS: m/z (ESI): Found [M+1]^+^: 557.2576; C_32_H_37_N_4_O_3_S requires [M+1]^+^: 557.2581.

#### 3.3.3. *N*-(5-((3-(4-Nitrophenylsulfonamido)propyl)amino)-9*H*-benzo[*a*]phenoxazin-9-ylidene)-*N*-propylpropan-1-aminium Chloride **3c**

The reaction between 5-(dipropylamino)-2-nitrosophenol hydrochloride **1a** (0.062 g, 2.40 × 10^−4^ mol, 1.5 eq.), concentrated hydrochloric acid (0.055 mL), and **2c** (0.060 g, 1.60 × 10^−4^ mol, 1 eq.) (reflux time: 23 h) gave compound **3c** as a blue solid (0.023 g, 23%). Mp = 236.1–238.0 °C. R*f* = 0.82 (dichloromethane/methanol 95:5). FTIR (KBr 1%): *υ*_max_ 3410, 3190, 3055, 2962, 2935, 2876, 1640, 1589, 1547, 1529, 1495, 1453, 1436, 1383, 1331, 1293, 1239, 1161, 1136, 1100, 1059, 1011, 962, 888, 854, 820 cm^−1^. ^1^H NMR *δ*_H_ (CD_3_OD, 400 MHz) 1.08 (t, *J* = 7.2 Hz, 6H, N(CH_2_CH_2_*CH_3_*)_2_), 1.83 (q, *J* = 7.4 Hz, 4H, N(CH_2_*CH_2_*CH_3_)_2_), 2.06 (t, *J* = 6.6 Hz, 2H, NHCH_2_*CH_2_*CH_2_NHSO_2_), 3.18 (t, *J* = 6.4 Hz, 2H, NHCH_2_CH_2_*CH_2_*NHSO_2_), 3.62–3.67 (m, 4H, N(*CH_2_*CH_2_CH_3_)_2_), 3.77 (t, *J* = 6.8 Hz, 2H, NH*CH_2_*CH_2_CH_2_NHSO_2_), 4.60 (broad s, 2H, 2 × NH), 6.91 (s, 1H, H-6), 6.98 (s, 1H, H-8), 7.36 (d, *J* = 9.6 Hz, 1H, H-10), 7.86 (t, *J* = 7.6 Hz, 1H, H-3), 7.93–7.99 (m, 2H, H-2 and H-11), 8.08 (d, *J* = 8.8 Hz, 2H, 2 × Ar-H *m*-NO_2_), 8.33 (d, *J* = 8.8 Hz, 2H, 2 × Ar-H *o*-NO_2_), 8.36 (d, *J* = 8.4 Hz, 1H, H-4), 8.98 (d, *J* = 8.0 Hz, 1H, H-1) ppm. ^13^C NMR *δ*_C_ (CD_3_OD, 100 MHz) 11.42 (N(CH_2_CH_2_*CH_3_*)_2_), 21.81 (N(CH_2_*CH_2_*CH_3_)_2_), 30.76 (NHCH_2_*CH_2_*CH_2_NHSO_2_), 41.52 (NHCH_2_CH_2_*CH_2_*NHSO_2_), 42.90 (NH*CH_2_*CH_2_CH_2_NHSO_2_), 54.63 (N(*CH_2_*CH_2_CH_3_)_2_), 94.36 (C-6), 97.23 (C-8), 117.32 (C-10), 123.82 (C-4), 124.75 (Ar-C), 125.45 (2 × Ar-C *o*-NO_2_), 125.72 (C-1), 129.44 (2 × Ar-C *m*-NO_2_), 130.96 (C-3), 132.30 (Ar-C), 132.76 (Ar-C), 133.06 (C-2), 134.27 (C-11), 134.89 (Ar-C), 147.60 (Ar-C), 149.93 (Ar-C), 151.35 (Ar-C), 153.33 (Ar-C), 156.46 (C-9), 159.31 (C-5) ppm. HRMS: m/z (ESI): Found [M+1]^+^: 588.2269; C_31_H_34_N_5_O_5_S requires [M+1]^+^: 588.2275.

#### 3.3.4. *N*-(5-((3-(Propylsulfonamido)propyl)amino)-9*H*-benzo[*a*]phenoxazin-9-ylidene)propan-1-aminium Chloride **3d**

The reaction between 5-(propylamino)-2-nitrosophenol hydrochloride **1b** [37] (0.147 g, 8.19 × 10^−4^ mol, 1.5 eq.), concentrated hydrochloric acid (0.294mL), and **2a** (0.167 g, 5.46 × 10^−4^ mol, 1 eq.) (reflux time: 18 h) gave compound **3d** as a blue solid (0.027 g, 39%). Mp = 193.4–195.4 °C. R*f* = 0.55 (dichloromethane/methanol 9:1). FTIR (KBr 1%): *υ*_max_ 3437, 3189, 2962, 2917, 2865, 1640, 1587, 1549, 1524, 1499, 1437, 1408, 1384, 1322, 1271, 1187, 1153, 1110, 1076, 1004, 948, 839, 816 cm^−1^. ^1^H NMR *δ*_H_ (CD_3_OD, 400 MHz) 1.09 and 1.10 (2 × t, *J* = 8.4 Hz, 6H, NHCH_2_CH_2_*CH_3_* and NHSO_2_CH_2_CH_2_*CH_3_*), 1.74–1.90 (m, 4H, NHCH_2_*CH_2_*CH_3_ and NHSO_2_CH_2_*CH_2_*CH_3_), 2.11 (quint, *J* = 6.8 Hz, 2H, NHCH_2_*CH_2_*CH_2_NHSO_2_), 3.10 (t, *J* = 7.6 Hz, 2H, NHSO_2_*CH_2_*CH_2_CH_3_), 3.29 (t, *J* = 6.4 Hz, 2H, NHCH_2_CH_2_*CH_2_*NHSO_2_), 3.39 (t, *J* = 7.2 Hz, 2H, NH*CH_2_*CH_2_CH_3_), 3.86 (t, *J* = 7.2 Hz, 2H, NH*CH_2_*CH_2_CH_2_NHSO_2_), 6.82 (d, *J* = 2.0 Hz, 1H, H-8), 7.02 (s, 1H, H-6), 7.14 (d, *J* = 7.2 Hz, 1H, H-10), 7.81–7.86 (m, 2H, H-3 and H-11), 7.94 (t, *J* = 7.6 Hz, 1H, H-2), 8.34 (d, *J* = 8.4 Hz, 1H, H-4), 8.93 (d, *J* = 7.6 Hz, 1H, H-1) ppm. ^13^C NMR *δ*_C_ (CD_3_OD, 100 MHz) 11.79 (NHCH_2_CH_2_*CH_3_*), 13.24 (NHSO_2_CH_2_CH_2_*CH_3_*), 18.41 (NHSO_2_CH_2_*CH_2_*CH_3_), 23.03 (NHCH_2_*CH_2_*CH_3_), 30.24 (NHCH_2_*CH_2_*CH_2_NHSO_2_), 41.33 (NHCH_2_CH_2_*CH_2_*NHSO_2_), 42.89 (NH*CH_2_*CH_2_CH_2_NHSO_2_), 46.43 (NH*CH_2_*CH_2_CH_3_), 54.45 (NHSO_2_*CH_2_*CH_2_CH_3_), 94.26 (C-6), 95.53 (C-8), 117.04 (C-10), 123.75 (C-4), 124.76 (Ar-C), 125.57 (C-1), 130.85 (C-3), 132.52 (Ar-C), 132.70 (Ar-C), 132.90 (C-2), 134.13 (Ar-C), 134.43 (C-11), 150.62 (Ar-C), 153.22 (Ar-C), 158.42 (C-9), 159.14 (C-5) ppm. HRMS: m/z (ESI): Found [M+1]^+^: 467.2115; C_25_H_31_N_4_O_3_S requires [M+1]^+^: 467.2111.

#### 3.3.5. *N*-(5-((3-(4-Methylphenylsulfonamido)propyl)amino)-9*H*-benzo[*a*]phenoxazin-9-ylidene)propan-1-aminium Chloride **3e**

The reaction between 5-(propylamino)-2-nitrosophenol hydrochloride **1b** (0.067 g, 3.10 × 10^−4^ mol, 2 eq.), concentrated hydrochloric acid (0.059 mL), and **2b** (0.055 g, 1.55 × 10^−4^ mol, 1 eq.) (reflux time: 16 h) gave compound **3e** as a blue solid (0.010 g, 8%). Mp > 300 °C. R*f* = 0.76 (dichloromethane/methanol 95:5). FTIR (KBr 1%): *υ*_max_ 3435, 2966, 2928, 2854, 1660, 1641, 1591, 1549, 1530, 1464, 1384, 1322, 1158, 1094, 971, 803 cm^−1^. ^1^H NMR *δ*_H_ (CD_3_OD, 400 MHz) 1.10 (t, *J* = 7.4 Hz, 3H, NHCH_2_CH_2_*CH_3_*), 1.79 (sext, *J* = 7.2 Hz, 2H, NHCH_2_*CH_2_*CH_3_), 2.05 (quint, *J* = 6.6 Hz, 2H, NHCH_2_*CH_2_*CH_2_NHSO_2_), 2.36 (s, 3H, Ph-*CH_3_*), 3.06 (t, *J* = 6.4 Hz, 2H, NHCH_2_CH_2_*CH_2_*NHSO_2_), 3.38 (t, *J* = 6.0 Hz, 2H, NH*CH_2_*CH_2_CH_3_), 3.80 (t, *J* = 7.2 Hz, 2H, NH*CH_2_*CH_2_CH_2_NHSO_2_), 6.81 (d, *J* = 2.4 Hz, 1H, H-8), 6.95 (s, 1H, H-6), 7.14 (dd, *J* = 9.2 and 2.0 Hz, 1H, H-10), 7.34 (d, *J* = 8.0 Hz, 2H, 2 × Ar-H *o*-CH_3_), 7.74 (dd, *J* = 8.4 and 2.0 Hz, 2H, 2 × Ar-H *m*-CH_3_), 7.80–7.86 (m, 2H, H-3 and H-11), 7.94 (t, *J* = 7.6 Hz, 1H, H-2), 8.32 (d, *J* = 8.0 Hz, 1H, H-4), 8.91 (dd, *J* = 8.2 and 1.0 Hz, 1H, H-1) ppm. ^13^C NMR *δ*_C_ (CD_3_OD, 100 MHz) 11.80 (NHCH_2_CH_2_*CH_3_*), 21.40 (Ph*CH_3_*), 23.05 (NHCH_2_*CH_2_*CH_3_), 29.53 (NHCH_2_*CH_2_*CH_2_NHSO_2_), 41.49 (NHCH_2_CH_2_*CH_2_*NHSO_2_), 42.98 (NH*CH_2_*CH_2_CH_2_NHSO_2_), 46.44 (NH*CH_2_*CH_2_CH_3_), 94.30 (C-6 and C-8), 112.02 (C-10), 123.76 (C-4), 124.80 (Ar-C), 125.56 (C-1), 128.07 (2 × Ar-C *m*-CH_3_), 130.77 (2 × Ar-C *o*-CH_3_), 130.86 (C-11), 132.48 (Ar-C), 132.66 (Ar-C), 132.89 (C-2), 134.11 (C-3), 134.42 (Ar-C), 138.50 (*C*-CH_3_), 144.81 (*C*-SO_2_), 153.09 (Ar-C), 158.41 (C-9), 159.09 (C-5) ppm. HRMS: m/z (ESI): Found [M+1]^+^: 515.2117; C_29_H_31_N_4_O_3_S requires [M+1]^+^: 515.2111.

#### 3.3.6. *N*-(10-Methyl-5-((3-(propylsulfonamido)propyl)amino)-9*H*-benzo[*a*]phenoxazin-9-ylidene)ethanaminium Chloride **3f**

The reaction between 5-(ethylamino)-4-methyl-2-nitrosophenol hydrochloride **1c** [60,61,62] (0.071 g, 3.80 × 10^−4^ mol, 1.5 eq.), concentrated hydrochloric acid (0.118 mL), and **2a** (0.067 g, 2.19 × 10^−4^ mol, 1 eq.) (reflux time: 20 h) gave compound **3f** as a blue solid (0.044 g, 48%). Mp = 141.2–143.5 °C. R*f* = 0.57 (dichloromethane/methanol 9:1). FTIR (KBr 1%): *υ*_max_ 3380, 3290, 2975, 2931, 2873, 1641, 1592, 1563, 1544, 1450, 1385, 1313, 1257, 1186, 1140, 1087, 1008, 965, 893, 822 cm^−1^. ^1^H NMR *δ*_H_ (CD_3_OD, 400 MHz) 1.05 (t, *J* = 7.6 Hz, 3H, NHSO_2_CH_2_CH_2_*CH_3_*), 1.32–1.39 (m, 3H, NHCH_2_*CH_3_*), 1.81 (sext, *J* = 7.6 Hz, 2H, NHSO_2_CH_2_*CH_2_*CH_3_), 2.05–2.15 (m, 2H, NHCH_2_*CH_2_*CH_2_NHSO_2_), 2.35 (s, 1H, *CH_3_*) 3.02–3.08 (m, 2H, NHSO_2_*CH_2_*CH_2_CH_3_), 3.20–3.30 (m, 2H, NHCH_2_CH_2_*CH_2_*NHSO_2_), 3.50–3.60 (m, 2H, NH*CH_2_*CH_3_), 3.80–3.87 (m, 2H, NH*CH_2_*CH_2_CH_2_NHSO_2_), 6.90 (s, 1H, H-8), 7.03 (s, 1H, H-6), 7.75 (s, 1H, H-11), 7.82 (t, *J* = 7.6 Hz, 1H, H-3), 7.92 (t, *J* = 7.6 Hz, 1H, H-2), 8.34 (d, *J* = 8.0 Hz, 1H, H-4), 8.96 (d, *J* = 8.0 Hz, 1H, H-1) ppm. ^13^C NMR *δ*_C_ (CD_3_OD, 100 MHz) 13.21 (NHSO_2_CH_2_CH_2_*CH_3_*), 14.12 (NHCH_2_*CH_3_*), 17.61 (*CH_3_*), 18.39 (NHSO_2_CH_2_*CH_2_*CH_3_), 30.21 (NHCH_2_*CH_2_*CH_2_NHSO_2_), 39.71 (NHCH_2_*CH_3_*), 41.32 (NHCH_2_CH_2_*CH_2_*NHSO_2_), 42.78 (NH*CH_2_*CH_2_CH_2_NHSO_2_), 54.44 (NHSO_2_*CH_2_*CH_2_CH_3_), 94.03 (C-6), 94.52 (C-8), 123.68 (C-4), 124.82 (Ar-C), 125.59 (C-1), 128.97 (Ar-C), 130.80 (C-3), 132.44 (Ar-C), 132.69 (C-2), 132.73 (C-11), 132.93 (Ar-C), 134.53 (Ar-C), 149.64 (C-10), 153.15 (Ar-C), 156.92 (C-9), 158.71 (C-5) ppm. HRMS: m/z (ESI): Found [M+1]^+^: 467.2109; C_25_H_31_N_4_O_3_S requires [M+1]^+^: 467.2111.

### 3.4. Photophysical Studies

Photophysical properties of benzo[*a*]phenoxazinium chlorides **3a–f** were determined in ethanol, ethanol acidified with TFA, water, and aqueous solutions at different pH values (3, 5, and 7.4), with concentrations between 10^−7^ and 10^−5^ M. These last solutions were prepared using boric acid, citric acid, and sodium phosphate buffers, or phosphate–saline buffer PBS (pH 7.4).

Fluorescence was measured at an angle of 90° to excitation incident radiation in quartz cells. The excitation wavelength was 590 nm for all compounds. The area below the fluorescence spectrum curve was determined, allowing the relative fluorescence quantum yield (*Φ*_F_) of the test compound to be calculated using Oxazine 1 as standard (*Φ*_F_ = 0.11 in ethanol [63]).

### 3.5. Antifungal Activity Assays

Minimum Inhibitory Concentration (MIC) of growth for compounds **3a–f** was determined using a broth microdilution method for the antifungal susceptibility testing of yeasts (M27-A3, CLSI—Clinical and Laboratory Standards Institute, Wayne, PA, USA). The yeast *S. cerevisiae* PYCC 4072 was used as a model organism. Cells were incubated at 30 °C in RPMI 1640 medium, buffered to pH 7.0 with 0.165 M morpholenepropanesulfonic acid (MOPS) buffer. Initial cell concentration was 2.25 × 10^3^ cells/mL. Stock solutions of the compounds were prepared in DMSO and a final dilution was carried out in an RPMI 1640 medium (DMSO concentrations of 0.5% per well). MIC values were determined using a microplate photometer, after 48 h of incubation, as the lowest concentration of drug that resulted in a growth inhibition over 80%, as compared with the growth observed in the control wells containing 0.5% DMSO. Each drug concentration was tested in triplicate and in three independent experiments.

### 3.6. Evaluation as Fluorescent Probes

*S. cerevisiae* W303-1A (MATa ura3-52 trp1∆2 leu2-3,112 his3-11 ade2-1), W303-1A Vba1-GFP (MATa ura3-52 trp1∆2 leu2-3,112 his3-11 ade2-1, pDF01-vba1-yeGFP(URA3)), BY4741 Sec66-GFP (MATa his3Δ1, leu2Δ0, met15Δ0, ura3Δ0), and 23344c jen1Δ Jen1-GFP (MATα ura3-52 jen1Δ, pGPD-jen1∆ct33-GFP(URA3)) were used to determine the intracellular distribution of the compounds. A sample of each culture was grown overnight at 30 °C and 120 rpm, in the respective medium: W303-1A and BY4741 Sec66-GFP (YEPD: 1% yeast extract, 2% peptone, 2% glucose); W303-1A Vba1-GFP and 23344c Jen1-GFP (SC: 2% glucose, 0.5% ammonium sulfate, 0.7% yeast nitrogen base w/o amino acids, 0.2% dropout mix, 0.01% histidine, and tryptophan, 0.02% leucine). For expression of the plasma membrane marker, 23344c Jen1-GFP was transferred to a medium with 2% lactic acid, 4 h before observation [58].

For general observation, W303-1A cells were stained with MIC concentration of the compounds for one hour, rinsed two times in PBS, and mounted on a slide for observation. To increase compound specificity and co-localization, W303-1A Vba1-GFP, BY4741 Sec66-GFP, and 23344c Jen1-GFP were stained with a low concentration of the compounds, 2.5 μM for one hour, rinsed two times in PBS, and mounted on a slide for observation.

The samples were analyzed on an Olympus BX6F2 fluorescence microscope, with appropriate filter cubes U-FDICT, ET-CY5-FAR-RED (exciter filter: 595–645 nm/emission filter: 665–715 nm) and TLV-U-FF-FITC (exciter filter: 455–495 nm/emission filter: 505–555 nm) with a 60× oil immersion objective. All treatment conditions were performed in three independent experiments.

## 4. Conclusions

A set of six new benzo[*a*]phenoxazinium chlorides functionalized with different sulfonamide groups at the 5-position were synthesized. These compounds display fluorescence emission in the NIR region, with maximum emission wavelengths (λ_emi_) between 642 and 687 nm, in the different tested solvents.

All compounds were found to exhibit inhibitory activity against *S. cerevisiae* PYCC 4072, with MIC values varying from 12.5 to >100 µM. The introduction of the sulfonamide group does not result in an improvement trend in the biological activity of the compounds in direct comparison with those previously described with identical core structures. From the point of view of the use of these compounds as fluorescent probes, this proves to be a positive aspect, as it is desirable that a probe does not affect the viability of a given organism. Indeed, the introduction of the sulfonamide group has quite positive effects on the fluorescent labeling compared with similar compounds [23,36].

Our results show that at a low concentration of 2.5 μM, these compounds are able to stain with high specificity the vacuolar membrane and/or the endoplasmic reticulum, and in the case of compounds **3d** and **3f**, even the plasma membrane. To the best of our knowledge, this is the first report in the literature of the synthesis of benzo[*a*]phenoxazinium chlorides capable of staining the plasma membrane. All compounds showed interesting fluorescent labeling patterns, but compounds **3d** and **3f** are remarkable for their labeling specificity. Their structural features, such as mono-alkylation at the 9-position and a propyl sulfonamide at the 5-position, are likely a key factor in this behavior. In addition, both compounds exhibit the highest fluorescence quantum yields of the set and are associated with the highest MIC values, making them promising bioimaging probes.

In this sense, this work contributes to the valorization of benzo[*a*]phenoxazinium chlorides as an alternative to commercial probes, as we were able to obtain very robust compounds that can be used in studies with fluorescence microscopy.

## Figures and Tables

**Table 1:** Photophysical data for benzo[*a*]phenoxazinium chlorides **3a**–**f**.

Compound		3a	3b	3c	3d	3e	3f
Ethanol	λ_abs_ (nm)|log *ε*	638|4.56	640|4.35 512|4.15	641|4.72	624|4.79	631|4.38	627|4.53
	λ_emi_ (nm)|*Φ*_F_	669|0.17	671|0.17	671|0.15	645|0.51	647|0.33	642|0.55
	Δλ (cm^−1^)	726	722	698	522	392	373
Acidified ethanol	λ_abs_ (nm)|log *ε*	639|4.58	640|4.73	641|4.78	626|4.77	633|4.50	627|4.60
	λ_emi_ (nm)|*Φ*_F_	670|0.18	669|0.19	671|0.19	645|0.52	646|0.32	643|0.58
	Δλ (cm^−1^)	724	677	698	470	318	397
Water	λ_abs_ (nm)|log *ε*	648|4.21	651|4.27	655|4.33	624|4.34	621|4.00	628|4.06
	λ_emi_ (nm)|*Φ*_F_	681|0.02	687|0.02	682|0.01	653|0.14	652|0.01	650|0.12
	Δλ (cm^−1^)	748	805	605	712	766	539
pH 3	λ_abs_ (nm)|log *ε*	647|4.46	651|4.57	653|4.38	625|4.66	635|4.28	625|4.49
	λ_emi_ (nm)|*Φ*_F_	677|0.03	680|0.04	684|0.02	654|0.18	657|0.10	651|0.24
	Δλ (cm^−1^)	685	655	694	709	527	639
pH 5	λ_abs_ (nm)|log *ε*	647|4.43	651|4.45	655|4.44	624|4.56	634|4.21	627|4.37
	λ_emi_ (nm)|*Φ*_F_	680|0.03	681|0.03	685|0.01	654|0.18	654|0.07	651|0.22
	Δλ (cm^−1^)	750	677	669	735	482	588
pH 7.4	λ_abs_ (nm)|log *ε*	647|4.36	652|4.01 537|3.92	656|4.37 550|4.17	624|4.49	634|4.02 526|3.70	621|4.40
	λ_emi_ (nm)|*Φ*_F_	676|0.03	683|0.01	686|0.02	652|0.15	655|0.06	649|0.20
	Δλ (cm^−1^)	663	696	667	688	506	695

**Table 2:** MIC and log *P* values of benzo[*a*]phenoxazinium chlorides **3a–f** against the yeast *S. cerevisiae* PYCC 4072.

Compound	3a	3b	3c	3d	3e	3f
MIC (µM)	25	12.5	25	50	>100	50
log *P*	2.38	3.49	3,00	2.32	3.43	2.20

## Data Availability

The data presented in this study are available on request from the corresponding author.

## Supplementary Materials

The following supporting information can be downloaded at: https://www.mdpi.com/article/10.3390/ijms24033006/s1.
