# Supplementary material for: Novel Benzo[a]phenoxazinium Chlorides Functionalized with Sulfonamide Groups as NIR Fluorescent Probes for Vacuole, Endoplasmic Reticulum, and Plasma Membrane Staining
Source: Int J Mol Sci. 2023 Feb 3;24(3):3006. doi: 10.3390/ijms24033006 (PMC9918004; doi:10.3390/ijms24033006)
Supplement: Supplementary file 1 [file ijms-24-03006-s001.zip › ijms-2140246-supplementary.pdf]

Supplementary Materials

Novel Benzo[*a*]phenoxazinium Chlorides Functionalized with Sulfonamide Groups as NIR Fluorescent Probes for Vacuole, Endoplasmic Reticulum, and Plasma Membrane Staining

João C. C. Ferreira [1,2,3,†], Rui P. C. L. Sousa [1,2,†], A. Preto [2,3], Maria João Sousa [2,3] and M. Sameiro T. Gonçalves [1,*]

[1] Centre of Chemistry (CQUM), Department of Chemistry, University of Minho, Campus of Gualtar, 4710-057 Braga, Portugal
[2] Centre of Molecular and Environmental Biology (CBMA), Department of Biology, University of Minho, Campus of Gualtar, 4710-057 Braga, Portugal
[3] Institute of Science and Innovation for Bio-Sustainability (IBS), University of Minho, Campus of Gualtar, 4710-057 Braga, Portugal
* Correspondence: msameiro@quimica.uminho.pt
† These authors contributed equally to this work.

1. Absorption and fluorescence spectra of compounds 3a-f

Absorption and fluorescence spectra of compounds **3a-f** in dry ethanol, acidified ethanol, water and aqueous solutions of pH 3, 5 and 7.4 are shown.

1. Absorption and fluorescence spectra of compounds 3a-f

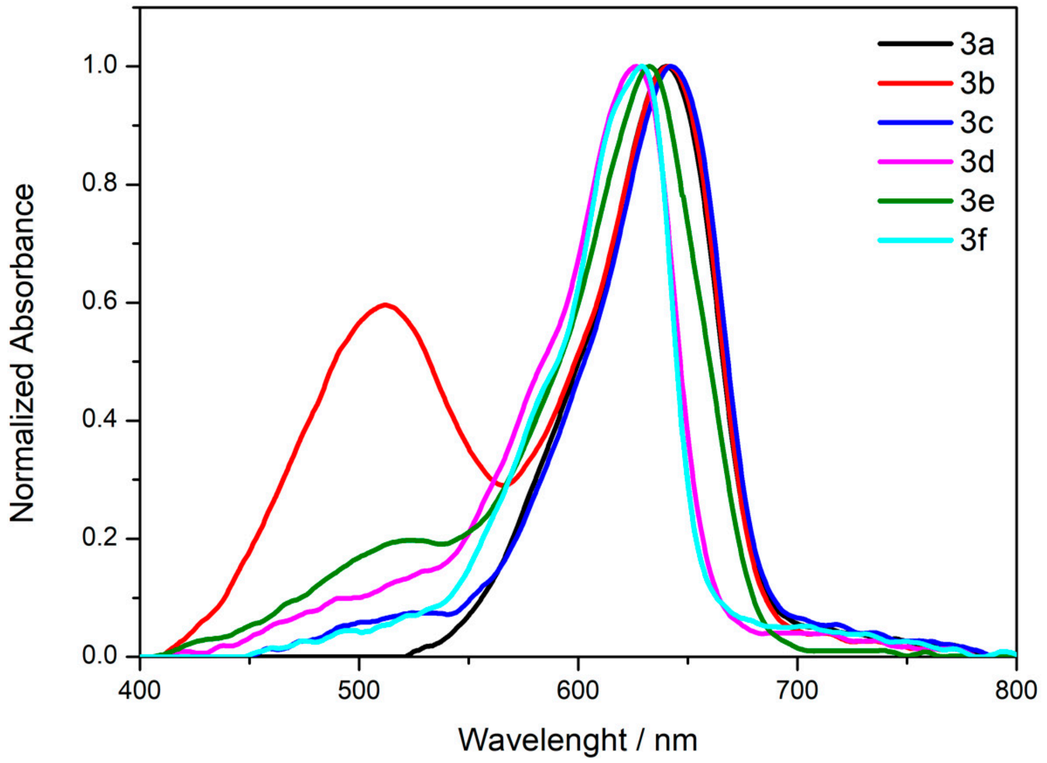

Figure S1 – Normalized absorption spectra of benzo[*a*]phenoxazinium chlorides **3a-f** in ethanol.

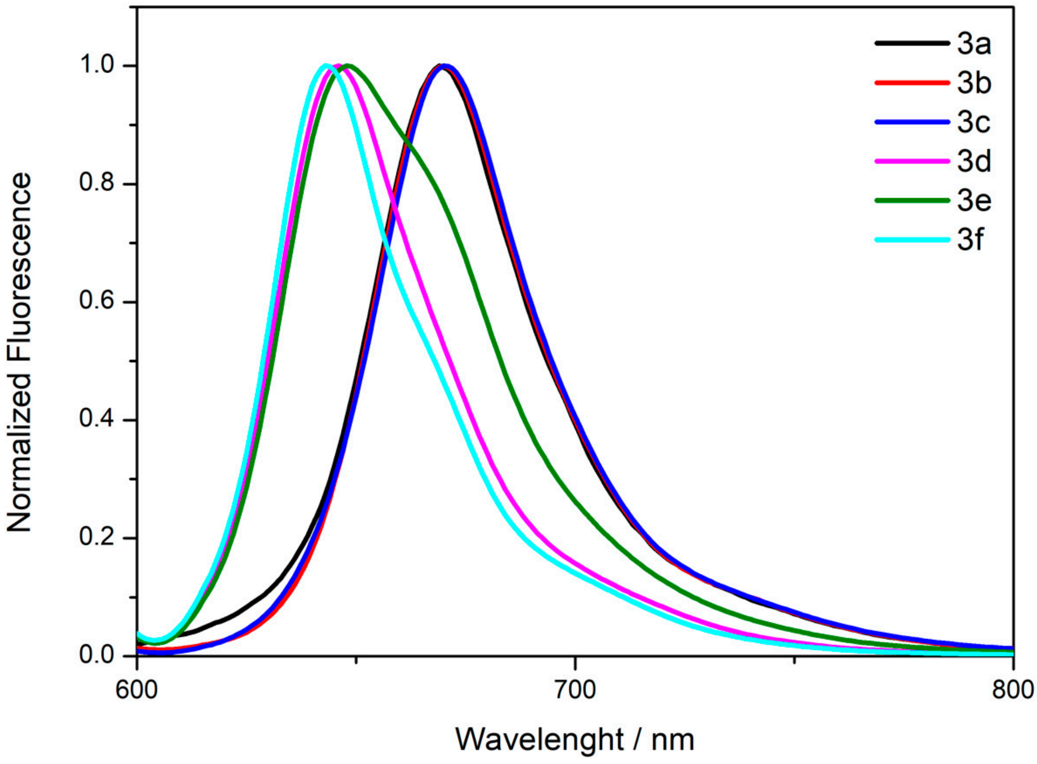

Figure S2 – Normalized emission spectra of benzo[*a*]phenoxazinium chlorides **3a-f** in ethanol, λ_{exc} = 590 nm).

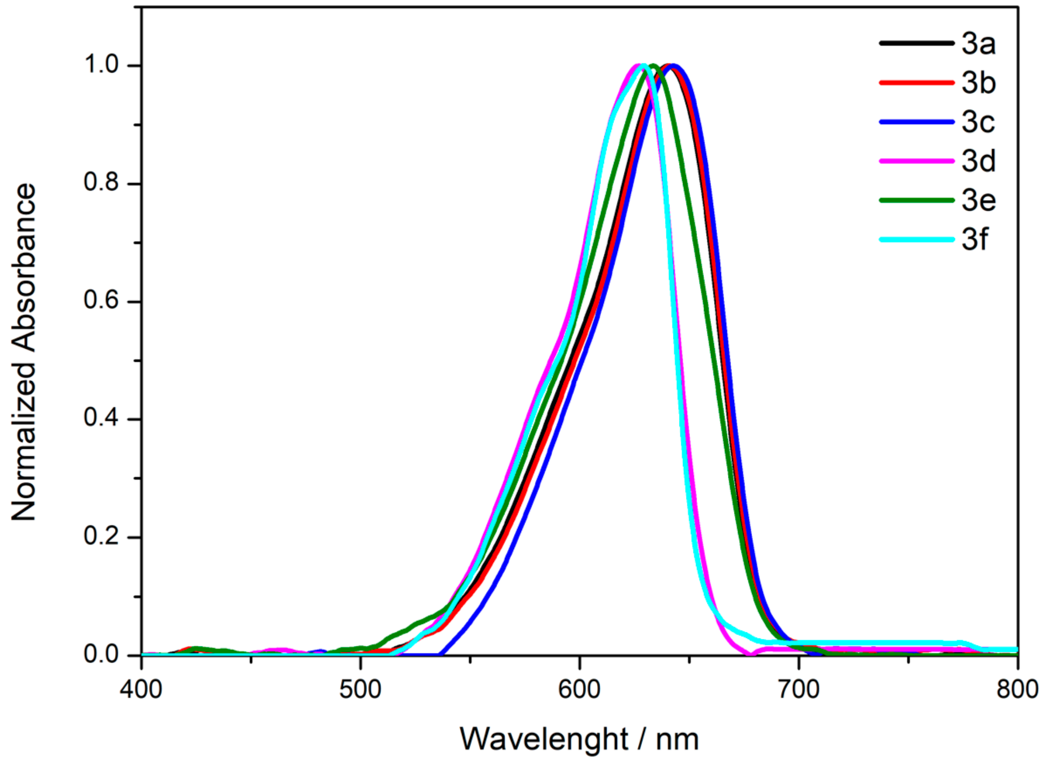

Figure S3 – Normalized absorption spectra of benzo[*a*]phenoxazinium chlorides **3a-f** in acidified ethanol.

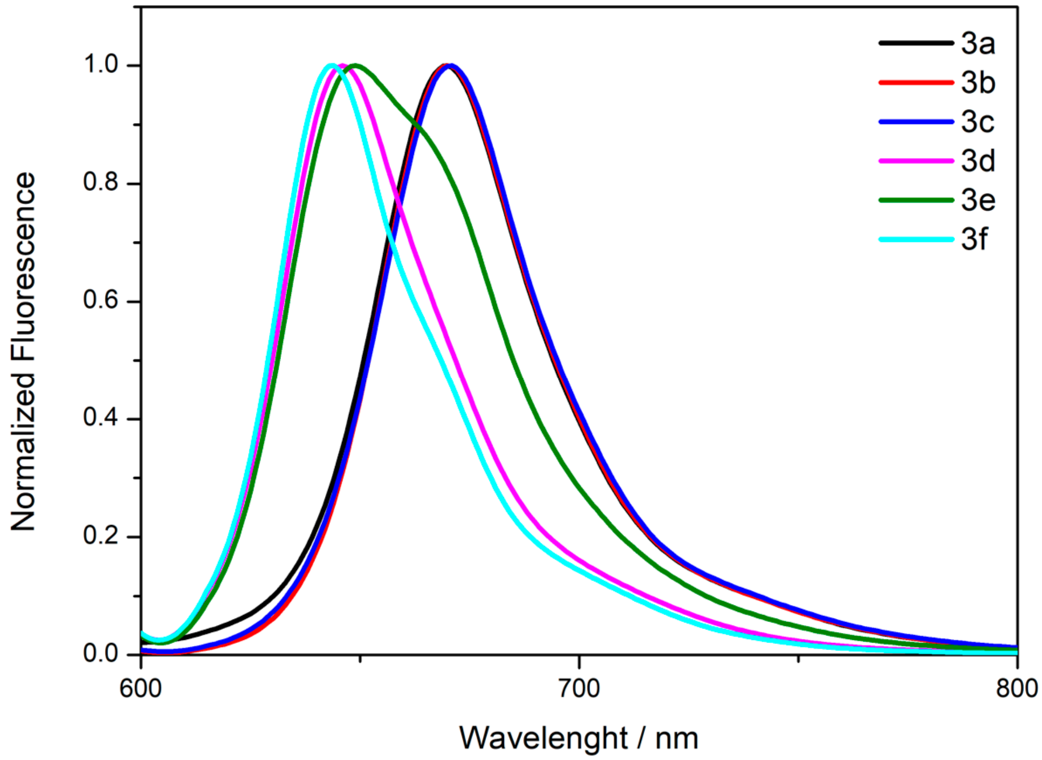

Figure S4 – Normalized emission spectra of benzo[*a*]phenoxazinium chlorides **3a-f** in acidified ethanol, λ_{exc} = 590 nm.

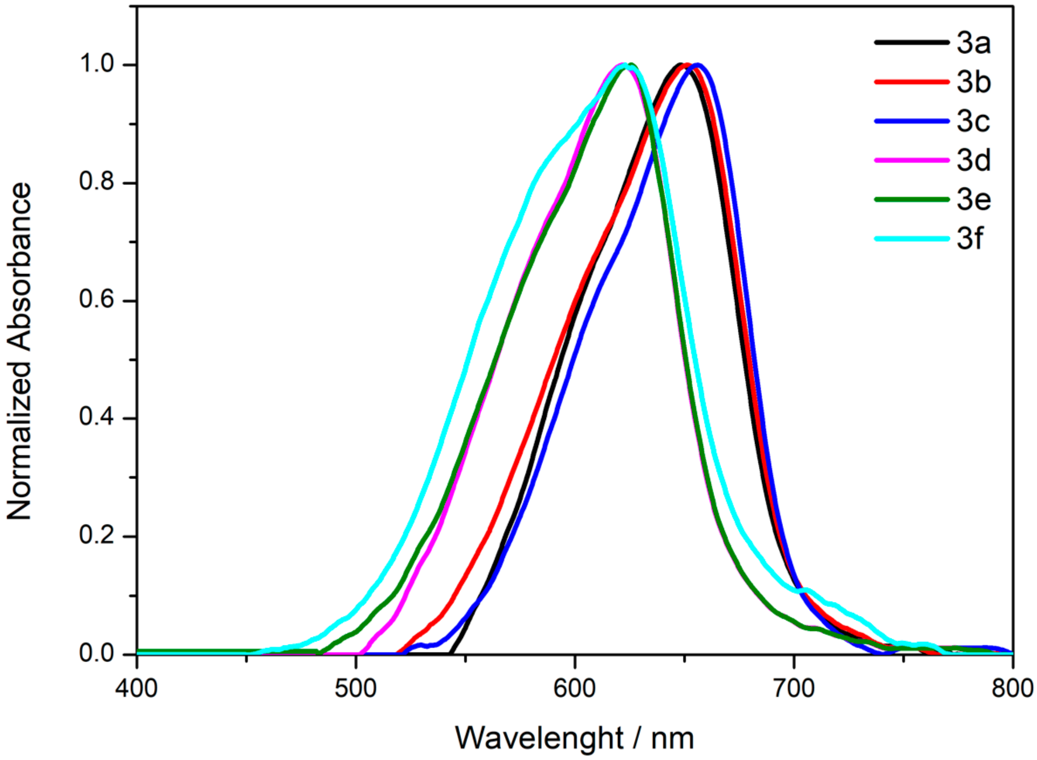

Figure S5 – Normalized absorption spectra of benzo[*a*]phenoxazinium chlorides **3a-f** in water.

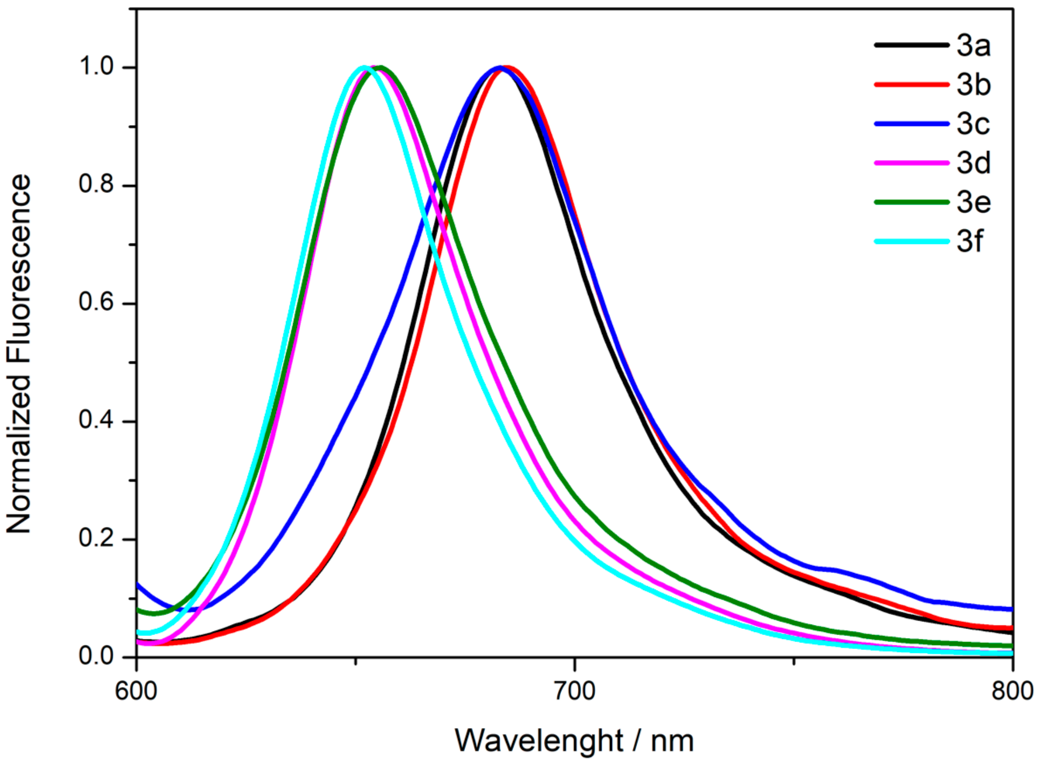

Figure S6 – Normalized emission spectra of benzo[*a*]phenoxazinium chlorides **3a-f** in water, λ_{exc} = 590 nm.

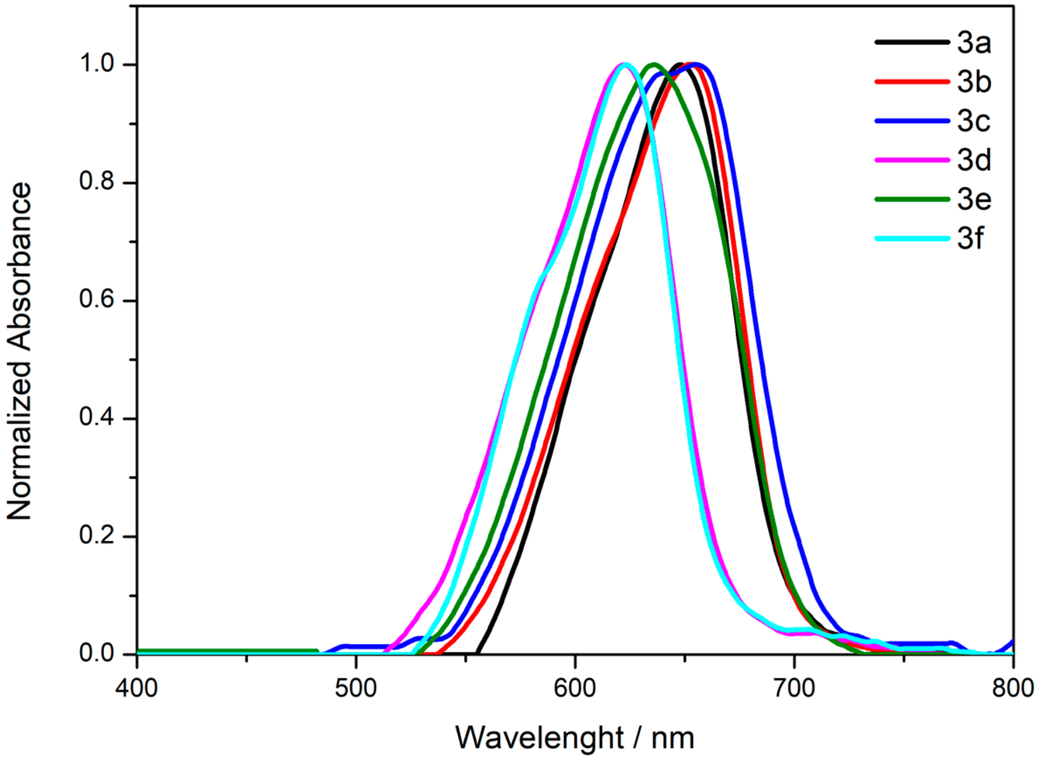

Figure S7 – Normalized absorption spectra of benzo[*a*]phenoxazinium chlorides **3a-f** in aqueous solution of pH=3.

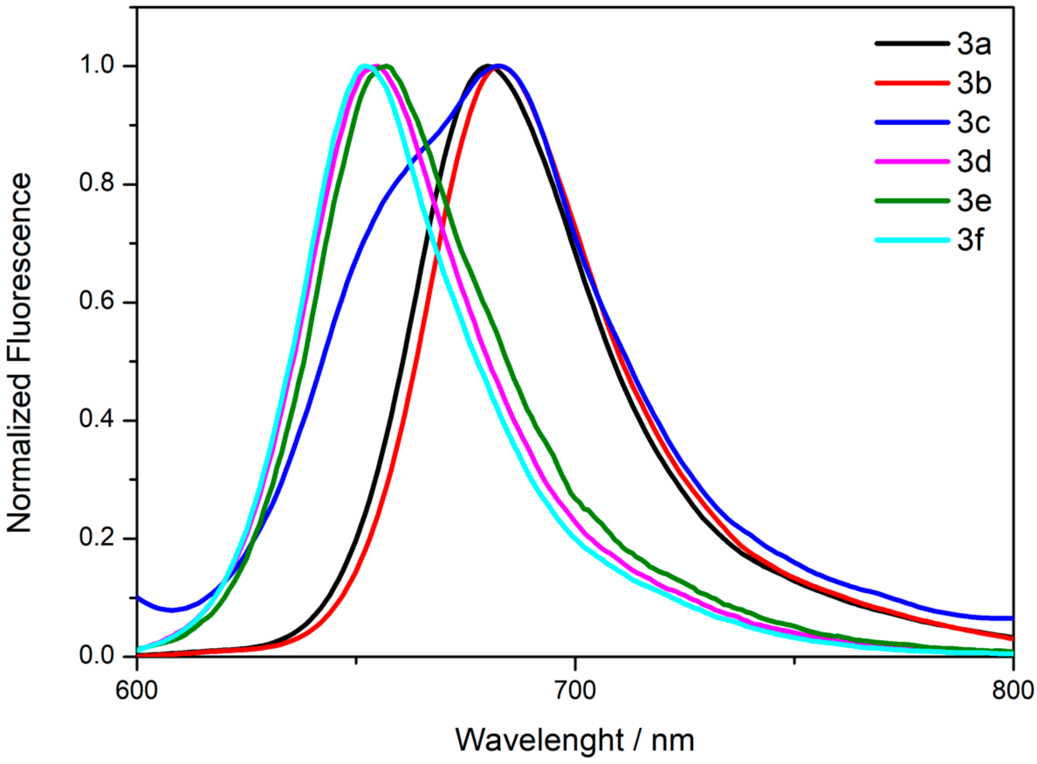

Figure S8 – Normalized emission spectra of benzo[*a*]phenoxazinium chlorides **3a-f** in aqueous solution of pH=3, λ_{exc} = 590 nm.

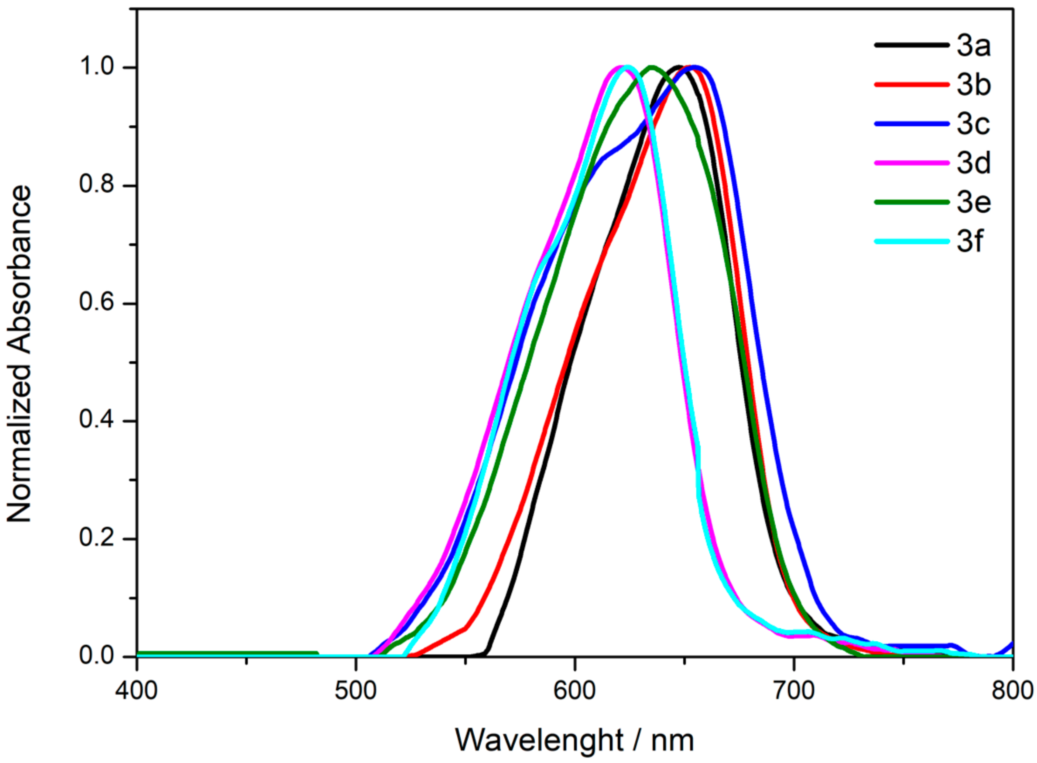

Figure S9 – Normalized absorption spectra of benzo[*a*]phenoxazinium chlorides **3a-f** in aqueous solution of pH=5.

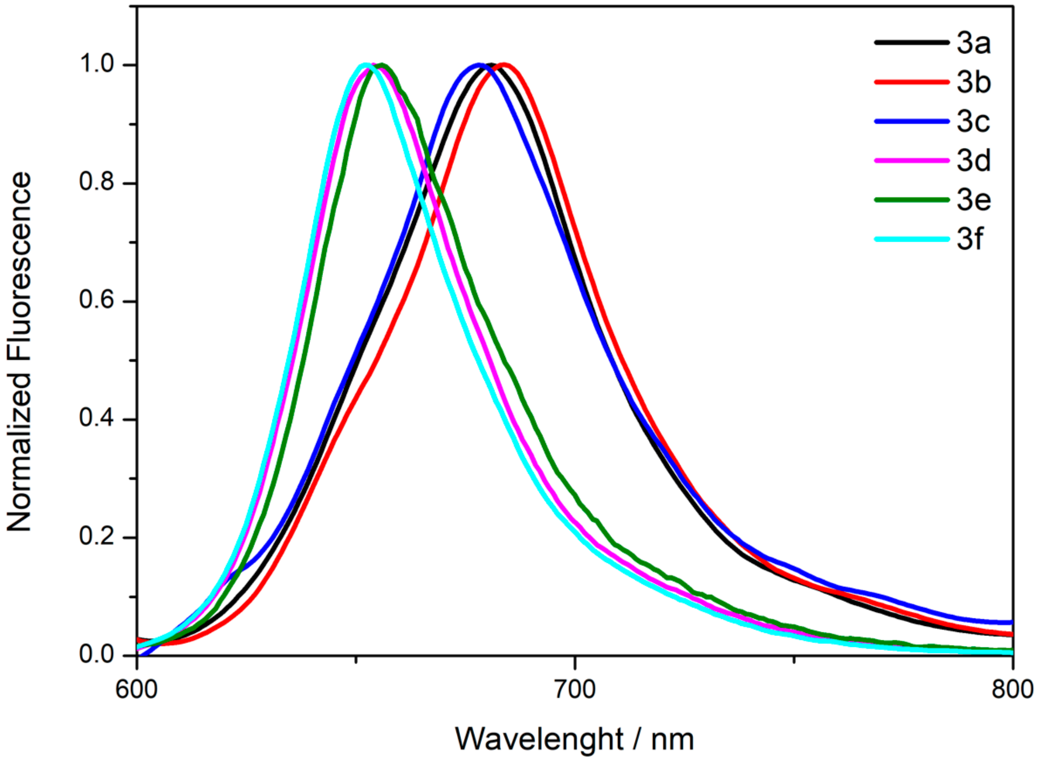

Figure S10 – Normalized emission spectra of benzo[*a*]phenoxazinium chlorides **3a-f** in aqueous solution of pH=5, λ_{exc} = 590 nm.

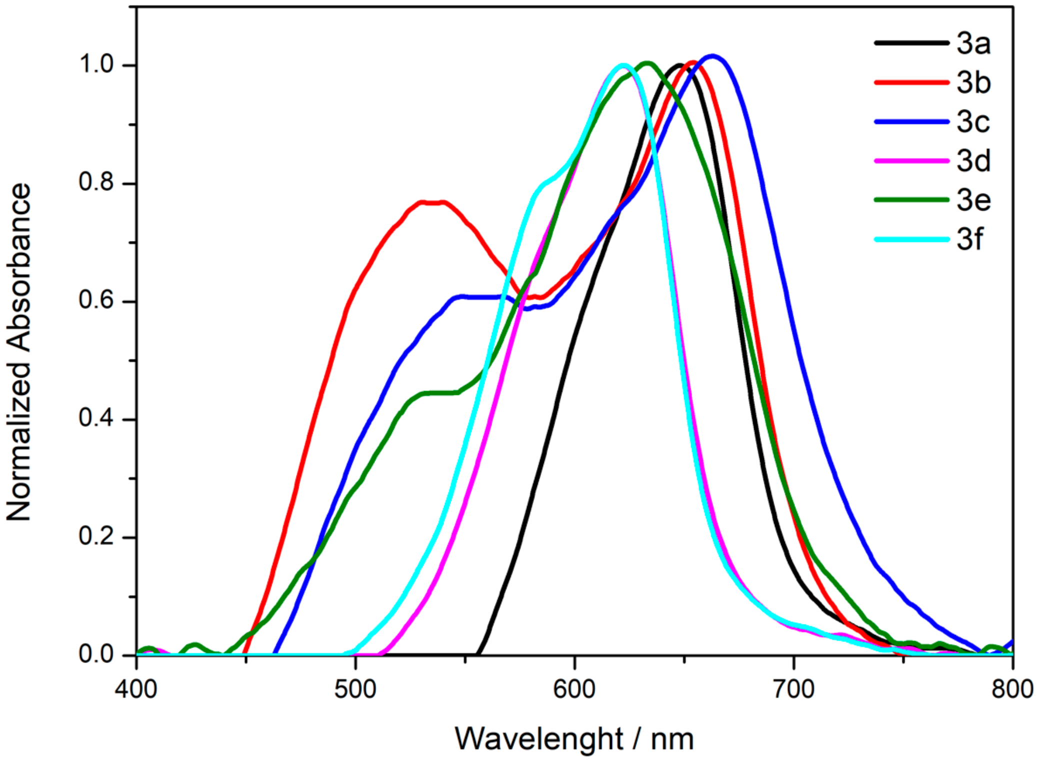

Figure S11 – Normalized absorption spectra of benzo[*a*]phenoxazinium chlorides **3a-f** in aqueous solution of pH=7.4.

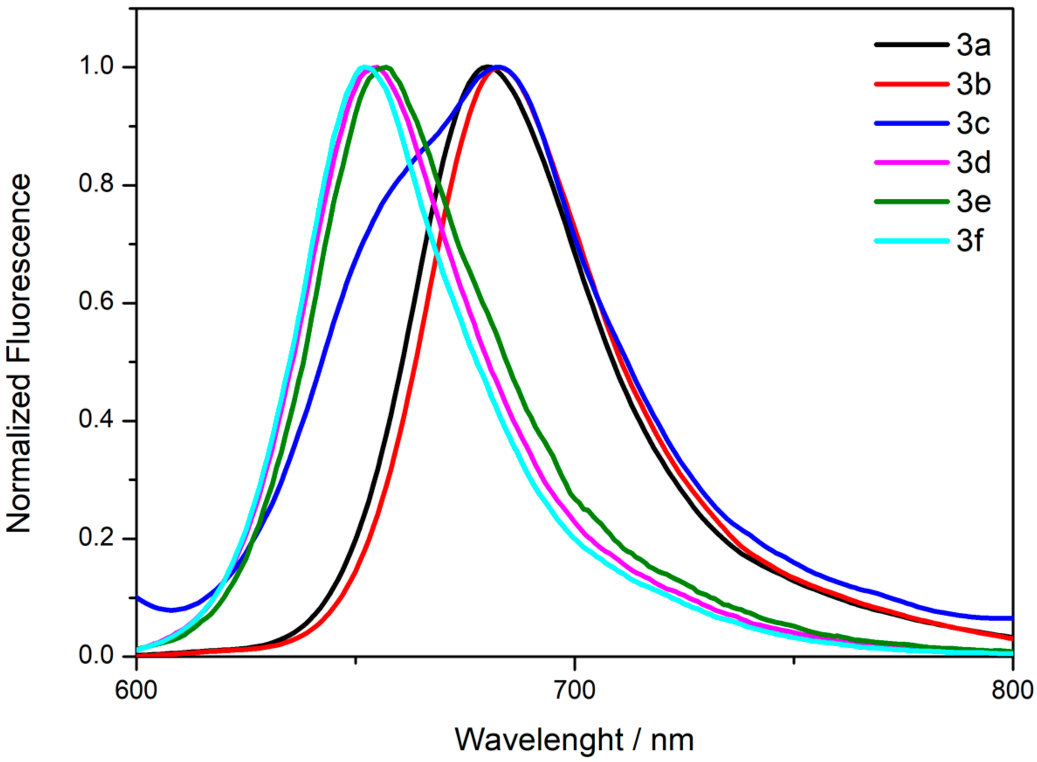

Figure S12 – Normalized emission spectra of benzo[*a*]phenoxazinium chlorides **3a-f** in aqueous solution of pH=7.4, λ_{exc} = 590 nm.
